# Biometric‐Tuned E‐Skin Sensor with Real Fingerprints Provides Insights on Tactile Perception: Rosa Parks Had Better Surface Vibrational Sensation than Richard Nixon

**DOI:** 10.1002/advs.202400234

**Published:** 2024-07-10

**Authors:** Senlin Hou, Qingyun Huang, Hongyu Zhang, Qingjiu Chen, Cong Wu, Mengge Wu, Chen Meng, Kuanming Yao, Xinge Yu, Vellaisamy A. L. Roy, Walid Daoud, Jianping Wang, Wen Jung Li

**Affiliations:** ^1^ Department of Mechanical Engineering City University of Hong Kong Hong Kong 999077 China; ^2^ Department of Industrial Engineering and Management School of Mechanical Engineering Shanghai Jiao Tong University Shanghai 200240 China; ^3^ State Key Laboratory of Mechanical Systems and Vibration Shanghai Jiao Tong University Shanghai 200240 China; ^4^ Hong Kong Centre for Cerebro‐cardiovascular Health Engineering (COCHE) Hong Kong 999077 China; ^5^ Department of Biomedical Engineering City University of Hong Kong Hong Kong 999077 China; ^6^ School of Science and Technology Hong Kong Metropolitan University Hong Kong 999077 China; ^7^ Department of Computer Science City University of Hong Kong Hong Kong 999077 China

**Keywords:** fingerprint pattern types, graphene oxide, manual fabric texture recognition, mechanoreceptors, tactile sensors

## Abstract

The dense mechanoreceptors in human fingertips enable texture discrimination. Recent advances in flexible electronics have created tactile sensors that effectively replicate slowly adapting (SA) and rapidly adapting (RA) mechanoreceptors. However, the influence of dermatoglyphic structures on tactile signal transmission, such as the effect of fingerprint ridge filtering on friction‐induced vibration frequencies, remains unexplored. A novel multi‐layer flexible sensor with an artificially synthesized skin surface capable of replicating arbitrary fingerprints is developed. This sensor simultaneously detects pressure (SA response) and vibration (RA response), enabling texture recognition. Fingerprint ridge patterns from notable historical figures – Rosa Parks, Richard Nixon, Martin Luther King Jr., and Ronald Reagan – are fabricated on the sensor surface. Vibration frequency responses to assorted fabric textures are measured and compared between fingerprint replicas. Results demonstrate that fingerprint topography substantially impacts skin‐surface vibrational transmission. Specifically, Parks' fingerprint structure conveyed higher frequencies more clearly than those of Nixon, King, or Reagan. This work suggests individual fingerprint ridge morphological variation influences tactile perception and can confer adaptive advantages for fine texture discrimination. The flexible bioinspired sensor provides new insights into human vibrotactile processing by modeling fingerprint‐filtered mechanical signals at the finger‐object interface.

## Graphical abstract

Inspired by biological sensory systems, a wearable flexible electronic skin sensor is developed that allows replicated human fingerprint structures as the sensor's interface surface to investigate the influence of dermatoglyphic structures on tactile signals. Furthermore, this tactile sensor has achieved the manual identification of surface structures with different interridge distances and fabric textures with different patterns.

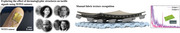

## 1 Introduction

Haptic feedback from the skin, which is one of several human sensing mechanisms, plays a vital role in our interactions with the environment,^[^ ^1^ ^]^ especially when performing delicate tasks, such as sharpening pencils, sewing clothes, and identifying the texture of fabrics. These tasks rely heavily on the highly developed tactile sensitivity of the fingertips. The process whereby mechanical signals (such as pressure or vibration) acting on the fingertip are conveyed to specific receptors through conditioning of the fingerprint and subsequently translated into neurological signals is known as fingertip tactile sensing.^[^ ^2^, ^3^ ^]^ Because fingertips have fingerprint structures and denser mechanoreceptors under the skin than other body skin areas, the fingerprint structure at a sub‐millimeter scale can differentiate patterns with a minimum interridge distance of 760 nm,^[^ ^4^ ^]^ swiping speed, environmental humidity, and fingerprint structure all affect test results.^[^ ^5^, ^6^ ^]^ In addition, various tactile sensations caused by fingerprint differences have attracted researchers’ attention, such as the modulating effect of the fingerprint ridge on the vibrational signals generated by touch.^[^ ^3^ ^]^ Moreover, interestingly, the ability of women to resolve finer surface details than men can be attributed to the differences in their dermatoglyphic structures.^[^ ^7^ ^]^

Inspired by human fingerprints, researchers have developed haptic sensors to study the role of fingerprints and to devise high‐precision artificial haptic systems for robotics, prosthetic limbs, and virtual reality scenarios.^[^ ^3^, ^8^, ^9^, ^10^, ^11^, ^12^, ^13^ ^]^ Haptic sensing systems based on accelerometers^[^ ^6^ ^]^ and Hall effect sensors^[^ ^14^ ^]^ are capable of precisely identifying tiny surfaces and textures on fabric surfaces. However, these stiff sensing technologies are incompatible with flexible surfaces. Rapid advances in flexible electronics technology have enabled researchers to employ piezoresistive,^[^ ^15^, ^16^, ^17^, ^18^ ^]^ capacitive,^[^ ^19^, ^20^, ^21^, ^22^ ^]^ and electric double layer (EDL)^[^ ^21^, ^23^ ^]^ technologies to construct flexible tactile sensors bearing microstructures. These sensors can be used to replicate slowly adapting (SA) mechanoreceptors that respond to pressure and touch. Additionally, tactile sensors based on piezoelectric^[^ ^24^, ^25^, ^26^ ^]^ and triboelectric techniques^[^ ^27^, ^28^ ^]^ have been developed to replicate rapidly adapting (RA) mechanoreceptors that respond to high‐frequency vibrations. Multi‐layer sensors^[^ ^29^, ^30^, ^31^, ^32^, ^33^, ^34^ ^]^ have also been devised for the simultaneous simulation of SA and RA mechanoreceptors, enabling the detection of pressure and vibration and thus texture recognition. However, few studies have investigated the influence of dermatoglyphic structures on tactile signals. Fingertip dermatoglyphic patterns are formed during the early stages of embryonic development under the influence of genetic factors,^[^ ^35^ ^]^ so fingerprint patterns have been extensively studied for disease‐related risk assessment and serve as a basis for the initial identification of certain limb and brain developmental abnormalities.^[^ ^36^, ^37^, ^38^ ^]^ The filtering effect of fingerprint ridges on the frequency of frictional vibration also affects our everyday tactile perception.^[^ ^3^ ^]^ Therefore, it is necessary to study the influence of the fingerprint's ridge structure on tactile signals.

Capacitive pressure sensors have been extensively studied due to their high structural stability, low power consumption, temperature‐independent, and rapid dynamic response.^[^ ^39^ ^]^ However, one drawback is that the deformation induced in the dielectric layer by an external force tends to be rather weak. As a result, the sensitivity of such capacitive pressure sensors tends to be significantly limited. Integrating microstructures such as micropyramids,^[^ ^21^, ^40^, ^41^ ^]^ micropillars,^[^ ^42^, ^43^, ^44^ ^]^ and microgrooves^[^ ^45^ ^]^ into the dielectric layer has proven effective for enhancing the sensitivity of capacitive pressure sensors. These microstructures can be introduced through direct replication of surface topographies from sandpaper^[^ ^46^, ^47^ ^]^ or plant leaves,^[^ ^48^ ^]^ which offer low‐cost approaches. However, this method cannot customize the shape, size, or spacing of microstructures. Researchers have attempted to utilize silicone molds to produce orderly and uniform microstructures. But the fabrication process is reliant on complex, and expensive lithography‐based methods.^[^ ^21^, ^40^, ^49^ ^]^ Besides optimizing the structures of the dielectric layer, the appropriate choice of materials is also pivotal to sensor performance. Elastomeric dielectrics are commonly used in capacitive sensors to improve their sensitivity, such as polydimethylsiloxane (PDMS). However the inherent viscoelasticity of PDMS inevitably prolonged response/recovery time,^[^ ^40^ ^]^ which restricted their application scope. Various approaches have attempted to address those limitations, one promising approach involves utilizing novel materials with high dielectric constants as the dielectric layer in capacitive pressure sensors.^[^ ^39^ ^]^ Graphene oxide (GO) is a versatile derivative of graphene with good mechanical, thermal, and electrical properties.^[^ ^50^ ^]^ Graphene consists of 2D crystalline sheets, and oxygen‐containing hydrophilic functional groups are introduced at the basal planes and edges during the oxidation of graphene, the presence of these functional groups introduces structural irregularities, which hinder the transfer of electrons along the GO planes.^[^ ^51^, ^52^ ^]^ Due to its high relative dielectric constant and water‐soluble properties, GO is an ideal dielectric layer for high‐sensitivity capacitive sensors. Research on pressure sensors based on GO remains limited. Thus, GO shows promising prospects as an enhanced dielectric material for dielectric layer material.

To investigate the effect of dermatoglyphic structures on subcutaneous vibrational signals during dynamic texture recognition, we developed high‐sensitivity capacitive electronic skins with real fingerprint structures as external structures. 3D printing technology is used to efficiently replicate fingerprint structures of human subjects and to develop dielectric layer microstructure templates for improving tactile force sensitivity. The dielectric layer material is GO mixed with PDMS and parylene C was coated on the surface of the dielectric layer to increase the dielectric constant and improve the frequency response of the sensor. The relationship between the scanning speed, sample roughness, sensor fingerprint structure, and vibrational spectrum was explored through experiments. The results have proven that the fingerprint interridge distance and contacts during scanning affect tactile vibrational signals. The type of fingerprint pattern affects the vibrational amplitude generated by fingertip movement during texture recognition. Furthermore, by using a 3D‐printed‐based real fingerprint as the external structure of the sensor, we achieved manual surface texture recognition. To the best of our knowledge, this has not previously been achieved. Overall, we developed a wearable fingerprint electronic skin sensor (WFES sensor) that can be applied in humanoid robots, bionic prostheses, augmented reality, and other technologies.

## 2 Result

### 2.1 Design Concept of the Wearable Fingerprint Electronic Skin (WFES) Sensor

**Figure 1:**
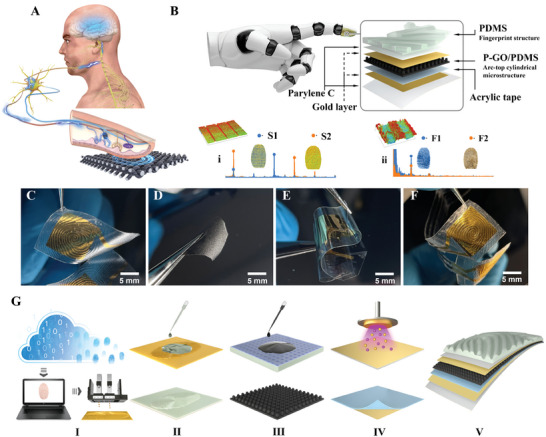
Design, construction, and fabrication of electronic skin inspired by fingertip. A) Perceptual mechanisms of the human haptic system. B) Schematic diagram of the layout of the WFES sensor and the study on the vibrational signal of different sensor structures using the WFES sensor, including i) The effect of the spacing of the stripe sensor structure on the tactile vibrational signal. ii) The effect of different fingerprint structures on the tactile vibrational signal. C–F) The optical image of the main components of the WFES sensor, from left to right are the fingerprint film electrode, P‐GO/PDMS dielectric layer, parylene electrode, and the assembled sensor. G) Illustration of the manufacturing process of the main components of the WFES sensor. I) Modeling of the fingerprint structure of the sensor. II) Fabrication of fingerprint film electrode. III) Fabrication of dielectric layer with arc‐top cylindrical structure. IV) Fabrication of parylene electrode film. V)Assembled WFES sensor.

When a fingertip moves across a textured surface, four functionally mechanoreceptors (the fast adapting receptors RA I and RA II, and slowly adapting receptors SA I and SA II) convert mechanical stimuli such as pressure, tension, and vibration into action potentials, which are transmitted through the nervous system to the cerebral cortex, where they produce sensory responses (**Figure** **1**A).^[^ ^53^, ^54^ ^]^ SA mechanoreceptors primarily perceive static stimuli and produce a continuous response that depends on the intensity of the stimulus, whereas RA mechanoreceptors perceive dynamic and produce a brief response at the beginning and end of a phase.

A schematic diagram of the layout of the WFES sensor is shown in Figure 1B, it is mainly composed of three parts, the fingerprint electrode film, the dielectric layer with the arc‐top cylindrical structure, and the parylene electrode film. For the fingerprint electrode film, considering the weak bonding between gold (Au) and PDMS, 2 µm of parylene C was plated onto the reverse side of the PDMS fingerprint film as a bonding layer between Au and PDMS to ensure the stability of the electrode. When GO is mixed into PDMS (GO/PDMS), the GO sheets hinder ion transfer within PDMS at low voltages, thereby increasing the effective dielectric constant, which is crucial for the sensitivity of sensors.^[^ ^51^ ^]^ The arc‐top cylindrical structure of the WFES sensor's dielectric layer comprises an array of arc‐top cylindrical structures with an adjacent spacing of 200 µm, which reduces the elastic modulus and increases the layer's pressure sensitivity.^[^ ^40^ ^]^ In addition, to prevent the viscoelasticity of PDMS, a 500 nm thick layer of parylene C was coated on the surface of the dielectric layer, the porous nature of PDMS allows for the diffusion of parylene C into the polymer^[^ ^55^ ^]^ (Figures S1 and S2, Supporting Information). This creates an interface between parylene and GO/PDMS (P‐GO/PDMS), benefiting from the low static friction coefficient of parylene C (0.29), which enhances the static and dynamic response of the sensor and enables to simulation of human mechanoreceptors to simultaneously acquire both high‐frequency and low‐frequency vibrations. In addition, parylene C on the surface of GO/PDMS materials will also significantly increase the dielectric constant (Figure S12, Supporting Information). The WFES sensor was used to study the vibrational signals of sensor structures, including i) the effect of the interridge distance of the sensor structure on the tactile vibrational signal; ii) the effects of different fingerprint structures on vibrational signals during fabric identification. Figure 1C–F shows the optical image of the fingerprint film electrode, P‐GO/PDMS dielectric layer, parylene electrode, and the assembled sensor, respectively. Figure 1G shows the fabrication processing of the WFES sensor which can be categorized into five different key steps as described below. I) Modeling of the fingerprint structure of the sensor by obtaining fingerprint pictures from the Internet or using a fingerprint scanner; then, a 3D printer was used to fabricate molds for the fingerprint film and the arc‐top cylindrical structure of the dielectric layer. II) Fabrication of fingerprint film electrode. III) Processing of dielectric layer with arc‐top cylindrical structure. IV) Fabrication of parylene C electrodes. V) Acrylic type is used to complete the sensor assembly (for detailed fabrication processes see Methods and Figure S3, Supporting Information).

### 2.2 Characterization of the WFES Sensor

**Figure 2:**
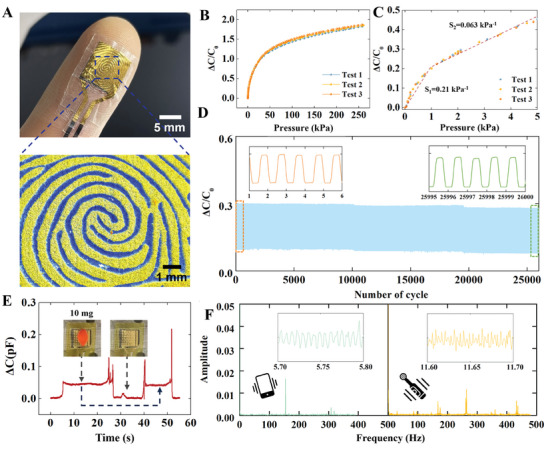
Characterization of the sensor performance including sensitivity, repeatability, resolution, and vibration response. A) Optical image of the WFES sensor attached to the fingertips and an optical 3D profile image of the fingerprint structure. B) Change in the sensor's capacitance over a pressure range of up to 250 kPa. C) Variation in capacitance over a pressure range of up to 5 kPa. D) The sensor's working stability was determined after 26 000 cycles from 0.5 to 3 kPa, inlets show the close‐up views at the beginning and end of the test. E) Response to the placement and removal of a petal on top of the sensor. F) Vibrational spectra of a smartphone and an electric toothbrush; insets show the time‐domain signal of the test.

A series of tests were conducted to evaluate the performance of this WFES sensor. The processing method replicates real fingerprint morphology to the maximum extent possible (**Figure** **2**A). Normal fingertip touch and grip require pressures ranging from 0 to 10 kPa and 10–100 kPa, respectively,^[^ ^32^ ^]^ so the test pressure range was 0–250 kPa. The pressure applied to the device was continually increased from 0 to 250 kPa using a MARK‐10 Series 5 Force Gauge (Mark‐10 Corp; Copiague, NY), and the associated change in capacitance was continuously recorded using an LCR meter (HIOKI IM3570, Japan) in the 10 kHz at 1 V. The rate of change in capacitance with applied pressure is depicted in Figure 2B, in which each applied pressure and the maximum capacitance response are plotted point by point. The equation *S = δ(ΔC/C_0_)/δP* gives the sensitivity of the flexible sensor, where *C*_0_ is the initial capacitance (13 pF); *ΔC* is the relative change in capacitance (*C – C*_0_); and *P* is the applied pressure. The sensor's sensitivity was thus calculated from the tangent of the curve. Figure 2C shows that the capacitance increased with pressure from 0 to 5 kPa, and the sensitivity was 0.21 kPa^−1^(0‐1 kPa). During the repeatability and stability testing, after being subjected to 26 000 cycles of pressure from 0.5 to 3 kPa, the sensor's sensitivity to pressure was not significantly diminished (Figure 2D). The inset of Figure 2D shows that the signals at the beginning and end of the repeated experiment had very similar waveforms. To determine the sensor's detection limit, a petal (weight: 10 mg) was put on the sensor and then removed (Figure 2E), and the changes in capacitance of the sensor are conspicuous in response to the weight of the petal.

In the sensor response time test, manual pressing using a steel ruler yielded a notable capacitance change of ≈17% (ΔC/C = 17%). The WFES sensor demonstrated impressive response and recovery times of 15 and 13 ms, respectively (refer to Note S1 and Figure S4, Supporting Information). Although the manual response tests did not allow for faster presses, the WFES sensor showcased the ability to detect high‐frequency vibrational signals, such as those generated by electric toothbrushes and smartphones. Specifically, the sensor was affixed to the back of a smartphone (iPhone 12) and was able to detect the smartphone's 154 Hz vibrations (inset of Figure 2F). Moreover, the sensor was also attached to a desktop to detect the transmitted vibration from an electric toothbrush (PHILIPS, HX9352/04, vibration frequency: 31 000 bpm) fixed on the desktop. The typical peaks of the electric toothbrush's vibrations are 170, 265, and 436 Hz, respectively (Figure 2F). As the maximum detectable frequency of RA mechanoreceptors in human skin is 400 Hz,^[^ ^33^ ^]^ these results showed that the sensor can detect signals that cover the frequency range of skin high‐frequency vibrations. The ability to detect high‐frequency vibrational signals is attributed to the highly effective dielectric constant of the P‐GO/PDMS material and the reduction of the viscoelasticity of PDMS by the parylene coating (for the detailed description of the sensing mechanisms of the WFES sensor see Note S4, Supporting Information).

### 2.3 Effect of Scanning Speed and Roughness

**Figure 3:**
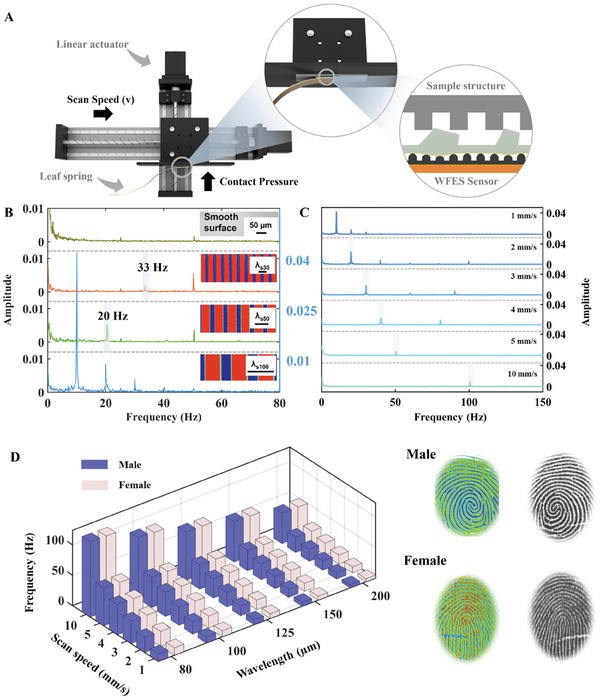
The effect of scanning speed and sample surface roughness on vibrational signals. A) Schematic diagrams of the experimental setup and WFES sensor deformation during scanning. B) The vibrational spectrum generated by the WFES sensor upon scanning Si microstrip (λ_s30_, λ_s50_, λ_s100_, and a smooth Si surface) with the fingerprint structure of Subject 1 at 1 mm s^−1^; insets show images of microstrip samples. C) Vibrational spectra at different scanning speeds. D) Relationship between the characteristic peaks in the vibrational spectrum, the interridge distance of the test sample, and the scanning speed with fingerprint patterns of a male subject (Subject 1) and a female subject (Subject 2) as the outer structure of the sensor, respectively.

The WFES sensor is a capacitive tactile sensor sensitive to vibrations. and capable of detecting surface roughness as it scans the surface of a sample. A sensor with a biomimetic fingerprint structure (right index finger of Subject 1, male, 26 years old; or pattern type: plain whorl; interridge distance: ≈600 µm) is attached to a leaf spring and fixed to the test bench (Reasons for choosing leaf springs to provide stable pressure and the pressure test of leaf spring see Note S5, Supporting Information). Samples are fixed to the surface of the linear actuator's fitting, where the linear actuator controls the sensor's movement relative to the samples (Figure S5, Supporting Information). The actuator's *x*‐axis was used to regulate the scanning speed (1–15 mm s^−1^) in the horizontal direction, and its *y*‐axis was used to control the pressure between the sensor and the sample. When the fingerprint structure of the sensor contacts with the sample surface, a shear force is generated that deforms the microstructure's internal dielectric layer (**Figure** **3**A). The capacitance fluctuated with the distance between the electrodes, producing a capacitance signal that was recorded by the capacitance acquisition board (PCAP04‐EVA‐KIT, ScioSense), which is more convenient for signal acquisition compared with the LCR meter.^[^ ^56^ ^]^ After the fast Fourier Transform (FFT), the frequency value with the highest amplitude was used as the characteristic frequency for measuring the surface roughness.

To demonstrate the sensor's capacity to distinguish tiny surfaces, Si microstrip with different interridge distances were scanned at a speed of 1 mm s^−1^ and a contact pressure of 2 kPa. The position of the characteristic peak decreased as the interridge distances of the scanned microstrip increased (Figure 3B). When the sensor scanned the Si microstrip with interridge distance λ_s30_ (for the detail of each Si microstrip see Figure S6, Supporting Information), the characteristic peak was at 33 Hz. When the sensor scanned the Si microstrip with interridge distances λ_s50_ and λ_s100_, the characteristic peak was at 20 and 10 Hz, respectively. When the sensor scanned a smooth structureless silicon surface, two distinct peaks generated by the stepper motor noise were observed, at 25 and 50 Hz. As has been determined,^[^ ^21^, ^31^ ^]^ the locations of the characteristic peaks are determined by the interridge distance of the measured surface and the scanning speed; that is, by the following equation (Equation. 1):

(1) $$f\;=\frac{v}{\lambda }$$

where *f* is the vibration frequency, *v* is the scanning speed, and λ is the sample's interridge distance. Thus, using the scanning speed and the interridge distances of the two Si microstrip (λ_s50_, λ_s30_) tested above, the peaks were calculated to be 20 and 33 Hz, respectively, which are consistent with the experimental results. This confirmed that the sensor was capable of reliably identifying patterns as small as 30 µm.

To investigate the influence of the scanning speed on the vibrational signal when the sensor's outer surface structure remains constant, various speeds (ranging from 1 to 10 mm s^−1^) were used to scan the microstrip with interridge distance λ_s100_. When the scanning speed was 1 mm s^−1^, the characteristic peak appeared at 10 Hz. When the scanning speed was doubled to 2 mm s^−1^, the frequency of the peak was doubled, as it appeared at 20 Hz. This demonstrated that the frequency of peaks increased linearly with the scanning speed (Figure 3C). Next, the microstrip with different interridge distances (λ_s80_, λ_s100_, λ_s125_, λ_s150,_ and λ_s200_.) were scanned with a fixed scanning speed and contact pressure (Figure 3D). The results show that the relationship between the sample interridge distance and the characteristic frequency is consistent with Equation (1). The experiment is repeated with the external structure of the sensor changed to a female fingerprint (right index of Subject 2, female, 27 years old; pattern type: double loop; interridge distance: ≈450 µm), the results were similar to those obtained with the male fingerprint structure.

### 2.4 Effect of Fingerprint Interridge Distance on the Vibrational Signal

Female fingertips can resolve finer surfaces than male fingertips, partly because female fingerprints and mechanoreceptors under females’ skin are denser than those of males.^[^ ^7^, ^57^, ^58^ ^]^ The aforementioned experimental results show that the WFES sensor performs exceptionally well in detecting fine surface roughness. Thus, this sensor can be used to investigate the effect of its fingerprint‐like structure on the vibrational signal.

**Figure 4:**
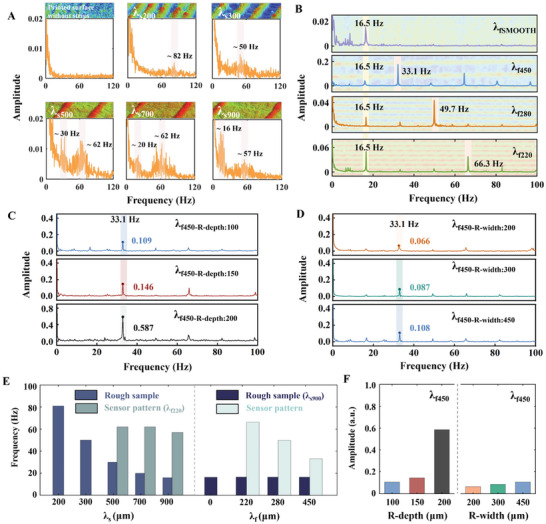
The effects of different sensor structures on captured vibrational signals. A) Vibrational spectra were acquired by the sensor with a striped structure (λ_f220_) by manually scanning various printed microstrips at an average speed of 15 mm s^−1^. B) The vibrational spectra were obtained by scanning the microstrip (λ_s900_) with WFES sensors having different interridge distances. C) The vibrational spectra were obtained by scanning the microstrip (λ_s900_) with WFES sensors having different ridge depths. D) The vibrational spectra were obtained by scanning the microstrip (λ_s900_) with WFES sensors having different ridge widths. E) Relationship between the interridge distance of the sensor structure, the interridge distance of the scanned sample, and the characteristic frequencies. F) Comparison of the effects of sensor structures with different ridge depths and ridge widths on vibrational signal amplitude.

The WFES sensor with a striped structure having an interridge distance λ_f220_ (for the detail of the sensor striped structure see Figure S7, Supporting Information) was attached to the outside surface of a rubber glove, and microstrip samples were manually scanned at different interridge distances (λ_s200_, λ_s300_, λ_s500_, λ_s700_, and λ_s900_, for the interridge distance detail of each printed microstrip sample see the Figure S8, Supporting Information) and at an average speed of 15 mm s^−1^, i.e., the finger swept ≈75 mm in 5 s at a constant speed. (video of manual identification of microstrips with different interridge distances see Movie S1, Supporting Information). The scanned samples were printed using a high‐precision 3D printer (DragonFly, Nano Dimension, Israeli), and optical images of the microstrip samples were placed on the frequency‐domain plot of each scan. When the interridge distance of the surface (λ_s200_ and λ_s300_) was less than or approximately equal to the interridge distance of the sensor structure (λ_f220_), the characteristic frequency peaks were proportional to the roughness of the sample surface (82 Hz for λ_s200_; 50 Hz for λ_s300_). When the interridge distance of surface (λ_s500_ and λ_s900_) was much greater than the interridge distance of the sensor structure, the measured frequencies included not only the characteristic peaks (30 Hz for λ_s500_, 20 Hz for λ_s700_, and 16 Hz for λ_s900_) associated with the sample surface roughness, but also characteristic peaks (62 Hz for λ_s500_, 62 Hz for λ_s700_, and 57 Hz for λ_s900_) attributable to the sensor structure. The position of the characteristic frequency peaks of the sensor structure did not change significantly with sample roughness, and so the characteristic frequency peak of the sensor structure could be calculated using Equation (1) (68.18 Hz for λ_f220_, for the detail of calculation, see Note S4, Supporting Information). When the sensor scanned a structureless sample at the same speed, the frequency‐domain signal distribution did not exhibit any distinctive peaks (**Figure** **4**A).

Fingerprint ridge density is one of the most important features of fingerprints, and female fingerprints tend to have greater ridge density than males. In a fingerprint pattern where ridges and valleys are interspersed with each other, the width of ridges varies from 100 to 300 µm and the ridge width distribution is not uniform in different regions of the fingerprint, the depth of fingerprint ridges is generally ≈50 µm.^[^ ^59^, ^60^, ^61^ ^]^ The ridge density can be evaluated by the interridge distances. To evaluate the relationship between the interridge distances of the exterior structure and the vibrational signal, four WFES sensors with striped structures having different interridge distances (λ_f220_, λ_f280_, λ_f450_, and smooth surface) were used to scan the microstrip sample with interridge distance λ_s900_ and at a fixed pressure (2 kPa) and speed (15 mm s^−1^). When four WFES sensors scanned the samples with interridge distance λ_s900_ at the same speed, the positions of the sample's surface roughness‐induced frequency peaks collected by the four sensor structures (λ_fSMOOTH_, λ_f450_, λ_f280,_ λ_f220_) were identical (16.5 Hz). However, the positions of the characteristic peaks generated by the sensor structure were not identical. That is, when a sensor structure with interridge distance λ_f450_ scanned microstrip with interridge distance λ_s900_, the characteristic peak induced by the sensor structure appeared at 33.1 Hz. Upon scanning by sensor structures with interridge distances λ_f280_ and λ_f220_, the induced characteristic peaks appeared at 49.7 and 66.3 Hz, respectively. Upon scanning with the sensor with a smooth surface structure, only one characteristic peak was induced and appeared at 16.5 Hz (Figure 4B). These results demonstrate that a nonlinear relationship exists between the peak frequency, the sample roughness, and the interridge distance of the sensor's surface structure. When the scanning speed and the sensor's structure were fixed, the curve of the sample interridge distance and characteristic frequency peak conforms to Equation (1). That is, as the interridge distance of the sample increased, the position of the characteristic peak decreased. When the scanning speed and sample structure were fixed, the characteristic peaks induced by the sample structures appeared at the same frequency, but the vibration generated by the sensor structures still conformed to Equation (1).

The impact of ridge depth on the vibrational signal was also tested. Three sensors with the same interridge distance λ_f450_ (for 2D profiles of sensor structures see Figure S14, Supporting Information) but different ridge depths (λ_f450‐R‐depth:100_, λ_f450‐R‐depth:150_ and λ_f450‐R‐depth:200_) were scanned on the sample surface with interridge distance λ_s900_ at the same speed (15 mm s^−1^) and pressure (2 kPa) (video of the scanning process see Movie S2, Supporting Information). The positions of the characteristic peaks induced by the sample and the sensor structure still appeared at 16.5 and 33.1 Hz, but the amplitude of the vibrational signal increased with the increase of the sensor ridge depth. In the time domain results (Figure S15, Supporting Information), the signal amplitude of the sensor with a ridge depth of 200 µm (λ_f450‐R‐depth:200_) was much larger than that of the sensors with ridge depths of 150 µm (λ_f450‐R‐depth:150_) and 100 µm (λ_f450‐R‐depth:100_). In addition, the dimensionless amplitude of the vibrational signal from the sensor with a ridge depth of 200 µm was 0.587, followed by the signal from the sensor with a ridge depth of 150 µm (0.146) and the sensor with a ridge depth of 100 µm (0.109), which was consistent with the time domain trend (Figure 4C). This is because, under the same pressure conditions, the sensor structure with a larger ridge depth produces more contact area (Figure S17, Supporting Information), thereby producing a larger contact deformation during scanning, resulting in a larger vibrational amplitude.

For the sensor scanning the sample surface with interridge distance λ_s900_ at different pressures at the same speed (15 mm s^−1^) also be tested, the time‐domain and frequency‐domain signals obtained from two sensor structures (λ_f450‐R‐depth:100_ and λ_f450‐R‐depth:150_) at different pressures showed that the positions of the characteristic peaks were consistent (16.5 and 33.3 Hz), the amplitude of the vibrational signal would increase with the increase of pressure, this is because the contact deformation between the sensor structure and the sample surface increases with increasing pressure. When the depth of the sensor structure reached 200 µm, the dimensionless amplitude of the vibrational signal was 0.587 at 2 kPa and 0.599 at 1.8 kPa, while the amplitude decreased when the pressure continued to increase or decrease (0.064 for 2.1 kPa, 0.267 for 1.7 kPa). For sensors with deeper ridge structures, even though the deformation of the sensor structure during scanning is larger compared to those with shallower ridges, the dielectric layer has been compressed to a greater extent in advance, which leads to reduced space for further deformation fluctuations of the dielectric layer. Consequently, the resultant vibrational signal amplitude is lower (0.064 for 2.1 kPa). As the pressure decreases, the compressible space of the dielectric layer is released, resulting in a vibrational signal with a larger amplitude. With a further decrease in pressure, the unstable contact between the sensor structure and sample surface causes a further decrease in the signal amplitude (0.267 for 1.7 kPa) (Figure S15, Supporting Information).

Three sensors with the same interridge distance (λ_f450_), and depth (100 µm) but different ridge widths were also fabricated to investigate the effect of variations in the ridge width on vibrational signals. The three sensors scanned the sample surface with interridge distance λ_s900_ at the same speed (15 mm s^−1^) and pressure (2 kPa). The positions of the characteristic peaks induced by the sample and the sensor structure in frequency‐domain signals remained at 16.5 and 33.1 Hz, respectively. The dimensionless amplitudes of the vibrational signals generated by the sensor structures with three different ridge widths were 0.066 (ridge width of 200 µm, λ_f450‐R‐width:200_), 0.087 (ridge width of 300 µm, λ_f450‐R‐width:300_) and 0.108 (ridge width of 450 µm, λ_f450‐R‐width:450_), respectively (Figure 4D; Figure S16, Supporting Information). The lesser impact of varying ridge widths on the amplitude of vibrational signals could be attributed to vibrational signals primarily originating from the initial contact between each sensor ridge and sample ridge during scanning. Therefore, under the same interridge distance, signals from ridges with different widths showed similar frequency and amplitude.

When the interridge distance of the sample was significantly greater than that of the sensor's outer structure, additional vibrations could be produced. Also, when the scanning speed and sample structure were fixed, the characteristic peaks induced by the sample structures appeared at the same frequency, but the vibration generated by the sensor structures conformed to Equations (S3) and (S4) (for the detail of the calculation process see Note S4, Supporting Information), as illustrated in Figure 4E. By comparing the experimental data collected using sensors with different ridge depths and widths, varying the ridge depth elicited larger changes to the signal amplitude than equivalent modifications to the ridge width (Figure 4F).^[^ ^62^ ^]^

In biological sensory systems, the process of tactile sensation is the result of the conversion of mechanical stimuli into action potentials by mechanoreceptors, the resulting signals are combined with information such as temperature and transmitted to the brain for integration. SA mechanoreceptors generate voltage spikes at different rates and patterns according to the intensity of the persistent stimulus, RA mechanoreceptors generate voltage spikes during the contact and release phases of the stimulus, and these signals are transmitted to the brain through the nerves to be integrated into the multi‐level tactile sensory information.^[^ ^29^, ^39^ ^]^ Biological mechanoreceptors can accurately sense signals of different frequencies and detect tactile stimuli such as pressure, temperature, and edges, and the results obtained after integration are more accurate than the information obtained by WFES sensors. However, the powerful high‐frequency vibration detection capability of the WFES sensor allows it to mimic the ability of human RA receptors to detect dynamic forces. The test results are consistent with the effects of fingerprints on texture recognition signals reported in psychophysics:^[^ ^62^ ^]^ The more textures fit together at the scale of the epidermal ridge, the more the average intensity in shear force, part of the friction, and resulting vibration at contact increases. Our scanning results herein suggest a new perspective and devices for this field: the signals generated during finger motion over rough surfaces contain not only information about surface roughness but also information related to fingerprint ridges.

### 2.5 Recognition of the Fabric Surface Texture

**Figure 5:**
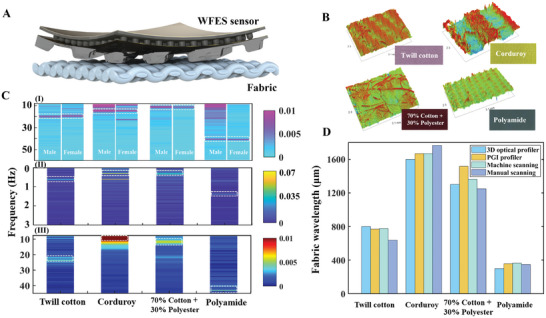
Fabric texture recognition test. A) Schematic diagram of the interaction between the WFES sensor and fabric. B) 3D fabric surface profile images of four fabrics (twill cotton, corduroy, 70% cotton + 30% polyester, and polyamide) were measured by the optical profilometer. C) Vibrational spectra were obtained by scanning four fabrics (twill cotton, corduroy, 70% cotton + 30% polyester, and polyamide) using a linear actuator (WFES sensor) I), PGI profilometer II), and manually scanning (WFES sensor) III). D) Comparison of fabric interridge distance measurement under four experimental conditions.

When a fingertip scans a fabric, the resulting mechanical stimulation induces mechanoreceptors under the skin to send electrical signals to the brain, which analyzes them to reach a conclusion regarding the fabric. The sensation of touching a fabric is often described with adjectives such as soft, smooth, dense, and warm. In addition, the amplitude and power intensity of the frequency spectrum of fabric scans are clearly correlated with specific wearability feelings.^[^ ^23^, ^25^ ^]^ Therefore, we used fabric scans to explore whether different types of fingerprint patterns produce different spectral signatures. **Figure** **5**A shows the fabric sensing process of the WFES sensor. The WFES sensor obtained vibrations by scanning the surface of the fabric and converting them to a capacitive signal. The interridge distance of the fabric was calculated from the characteristic peak position of the vibrational spectrum. Seven fabrics (corduroy, 70% cotton + 30% polyester, twill cotton, polyamide, silk, polyester, and hemp) were tested (Figure S9, Supporting Information).

To evaluate the accuracy of the WFES sensor's fabric recognition, four fabrics were selected for measurement using a phase grating interferometer (PGI) profilometer (Taylor Hobson, Form Talysurf Series 2) and an optical surface profilometer (Veeco/Wyko NT9300). The optical surface profilometer displayed a better 3D profile of the fabric surface than the PGI profilometer. First, the optical 3D contour images of twill cotton, corduroy, 70% cotton + 30% polyester, and polyamide are shown in Figure 5B. This indicates that four fabrics had regular features—polyamide, corduroy, 70% cotton + 30% polyester, and twill cotton—and their interridge distances were thus determined, using the scale, as ≈1500, 1300, 800, and 300 µm, respectively. Then, fabric samples were fixed to the surface of the linear actuator's fitting, and the relative movement between the sample and the surface structure (which was based on the fingerprint patterns of Subject 1) of the WFES sensor was controlled by the linear drive at a fixed pressure (2 kPa) and speed (15 mm s^−1^). The characteristic peak of corduroy is at 9 Hz, and its calculated interridge distance is 1666 µm. For polyamide, 70% cotton + 30% polyester, and twill cotton, the characteristic peaks are located at 41, 11, and 19.5 Hz, and the corresponding fabric interridge distance calculation results were 365, 1363, and 769 µm, respectively (Figure 5C‐I), darker color indicates higher amplitude. In the scanning results, the characteristic peak positions of the male and female fingerprint patterns matched perfectly but their amplitude differed. The female fingerprint sensor generated higher‐amplitude vibrations across the surfaces of polyamide, whereas the male fingerprint pattern generated higher‐amplitude vibrations across the surfaces of the corduroy and 70% cotton + 30% polyester.

The PGI profilometer's probe traversed the fabric surface at a speed of 0.5 mm s^−1^ during the measuring process, and the surface structure changed the signal vertically through the principle of leverage, after which the surface profile was measured. The characteristic peak of polyamide was at 1.4 Hz, and the calculated interridge distance λ_Polyamide_ is 357 µm. Corduroy, 70% cotton + 30% polyester, and twill cotton had characteristic peaks at 0.3 Hz (λ_Corduroy_ = 1,666 µm), 0.33 Hz (λ_7c3p_ = 1,515 µm), and 0.65 Hz (λ_Twill cotton_ = 769 µm), respectively (Figure 5C‐II). The measurement results of the WFES sensor are generally consistent with the results of the PGI surface profilometer. Specifically, the WFES sensor measurement results of corduroy and twill cotton are completely consistent with the measurement results of the profilometer. The surface features of 70% cotton + 30% polyester vary more smoothly in height between ridges and valleys than those of the other three fabrics, also, compared with the surfaces of corduroy and twill cotton, the surface of 70% cotton + 30% polyester has more fluff, which is spread during the sliding process to fill the height difference between the ridge and the valley, which cause large measurement deviation.

Attaching the same WFES sensor to the fingertip for manual scanning (the pressure was ≈5 kPa, and the average sliding speed was ≈15 mm s^−1^), the characteristic peaks of corduroy, 70% cotton + 30% polyester, twill cotton, and polyamide were at 8.5 Hz (λ_Corduroy_ = 1,764 µm), 12 Hz (λ_7c3p_ = 1250 µm), 20 Hz (λ_Twill cotton_ = 750 µm), and 35 Hz (λ_Polyamide_ = 428 µm), respectively (Figure 5C‐III). Compared with the PGI profilometer results, the measurement deviations for the four fabrics were 5.8%, 5.2%, 9.9%, and 19.9%, respectively. A comparison of the fabric interridge distance measurements under the four test conditions is shown in Figure 5D. When the scanning speed was kept constant, the fabric interridge distances detected by the WFES sensor were consistent with the PGI profilometer values. Due to the instability of the scanning speed, the manual test produced some deviation, but it could be used to identify different fabrics. In comparison with fabric sensory systems already reported, the WFES sensor system can manually recognize fabrics with high accuracy through wearable acquisition circuitry while simplifying the measurement system (Table S1, Supporting information).

### 2.6 Effect of Fingerprint Pattern Type on Tactile Signals in Fabric Recognition

**Figure 6:**
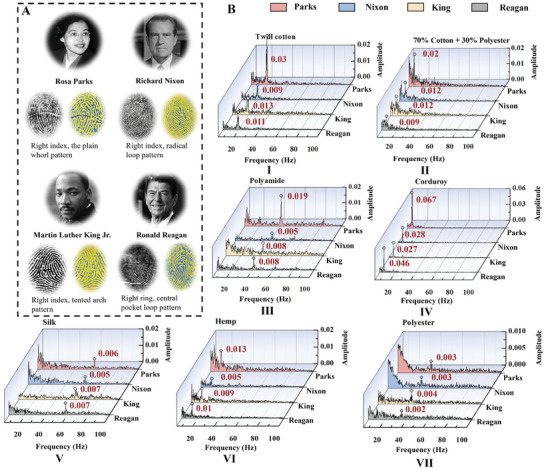
Testing the effect of fingerprint pattern type on fabric texture recognition signals. A) Fingerprints of four influential figures from the realms of politics and civil rights were chosen as samples for the research (Rosa Parks, Getty Images; Richard Nixon, Bachrach/Getty Images; Martin Luther King, Jr., Getty Images; Ronald Reagan, Universal History Archive/Getty Images).^[^ ^68^, ^69^, ^70^, ^71^ ^]^ B) Comparison of vibrational spectra obtained from WFES sensors of the famous political and civil rights leaders.

Human fingerprint patterns are not only used as an identification tool but are also considered to indicate talent, as people with outstanding achievements tend to have rare fingerprint patterns.^[^ ^63^ ^]^ Regular ridges and grooves on the finger surface form three main types of patterns: arches, loops, and whorls. To study the effect of fingerprint pattern type on vibrational spectra, we collected the published fingerprints of two former U.S. presidents and two prominent civil rights leaders as experimental samples.^[^ ^64^, ^65^, ^66^, ^67^ ^]^ Three types of fingerprint patterns were selected from their fingerprints to create WFES sensors and conduct fabric scanning experiments to fit real scenarios. The right index finger pattern (the plain whorl pattern) of the African–American civil rights activist Rosa Louise McCauley Parks; the right index finger pattern (tented arch pattern) of African American civil rights leader Martin Luther King, Jr.; the right index finger pattern (radical loop pattern; the frequency of this pattern is 0.98%) of the 37th president of the United States, Richard Milhous Nixon; and the right ring‐finger pattern (central pocket loop pattern) of the 40th president of the United States, Ronald Wilson Reagan, was selected as the study samples (**Figure** **6**A).

Four WFES sensors scanned seven fabrics at a fixed pressure (2 kPa) and speed (15 mm s^−1^), respectively. The results obtained from the four fingerprint patterns agree in terms of the frequency positions of the characteristic peaks, but the amplitudes are different (Figure 6B). The sensor with Parks’ fingerprint obtained higher‐amplitude vibrational signals on most fabric surfaces (e.g., twill cotton, 70% cotton + 30% polyester, polyamide, corduroy, and hemp) compared to sensors with other fingerprints. Based on the results of previous experiments, the interridge distance, the depth, and width of the fingerprint ridges, the pressure as well as the scanning direction all affect the amplitude of the vibrational signal (Figure 4; Figures S15 and S10, Supporting Information). When human fingers are used to recognize different surface textures, they continuously adjust their scanning speed and scanning direction to find the best tactile sensation, and fingerprints are a combination of curved ridges with different widths and depths, which can be adapted to scanning in all directions. Therefore, in the daily scanning process, the vibrational signals produced by different fingerprint patterns differ in amplitude but have a tiny effect on the vibrational frequency. However, further research is needed on the relationship between the vibrational amplitude and the fingerprint ridge contact.

## 3 Discussion

The tactile sense of humans is critical for sensing small variations in the environment and has long attracted research interest. The fingertip's dense tactile receptors enable it to discern tiny surface textures, however, the impact of fingerprints on tactile sensation still needs further research. In this study, inspired by fingerprints, we developed a unique WFES sensor to investigate fingerprint function and surface texture identification. The dielectric layer of the sensor was composed of a single‐layer GO and PDMS hybrid material. The electrode was a PDMS film with a biomimetic fingerprint structure. The sensor's high sensitivity (0.21 kPa^−1^ in the pressure range 0–1 kPa) and short response time (15 ms, ΔC/C = 17%) enabled it to distinguish microstrip with interridge distance as small as 30 µm. The sensor was used to explore the relationship between scanning speed, sample roughness, fingerprint interridge distance, and vibrational spectra. The frequency results of the microstrip samples test provide new perspectives on the two subcutaneous sensing mechanisms proposed in psychophysical studies. We used the published fingerprints of two U.S. presidents and two U.S. civil rights movement leaders to create WFES sensors and investigate, for the first time, the effect of different fingerprint pattern types on vibrational signals. In addition, manual recognition of fabrics and microstrips of different interridge distances was achieved with the WFES sensor attached to the fingertip, indicating great promise for the use of the sensor as artificial skin. Moreover, the combination with the rapidly developing skin hydrogel technology will further advance its development as an electronic skin.^[^ ^72^ ^]^

## 4 Experimental Section

### Fabrication of the Fingerprint Mold and Microstrip Samples

For the fabrication of the fingerprint mold, a fingerprint scanner (ZKTeco, China) was used to collect fingerprints, and then the mirrored fingerprint patterns were imported into CAD software and designed into fingerprint mold with a ridge depth of 50 µm. Next, the mold was imported into a 3D high‐precision inkjet light‐curing printer (DragonFly, Nano Dimension, Israeli) for fabricating. The microstrip samples were designed by CAD software and fabricated by the same 3D printer. The optical images of the printed fingerprint molds and microstrip samples are shown in Figures S7 and S8 (Supporting Information).

### Fabrication of the Fingerprint Electrode

The microstructures of the WFES sensor, including the fingerprint structure and the arc‐top cylindrical structure of the dielectric layer, were all fabricated using 3D printing technology, thus avoiding the use of complicated lithography‐based methods. For the fabrication of the fingerprint electrodes, first, the promotion solution is prepared (isopropyl alcohol (IPA), deionized water (DI), and A‐174 solution(Aladdin), proportion by volume, IPA: DI: A‐174 = 50:50:1), stir the solution with a clean stirring rod for 30 s and allow the solution to stand for at least 2 h, submerge the molds in the prepared promotion solution for 20 min and remove molds and air dry for 20 min, finally dry after rinsing with IPA. 1 µm of parylene C (Pressure: 15 mTorr, Furnace Set Point: 690 °C) was plated onto the mold (Figure S3‐I, Supporting Information) to help demolding in the subsequent steps. The PDMS film with a fingerprint structure was fabricated by spin coating (1000 rpm) PDMS onto the mold (Figure S3‐II, Supporting Information). Considering the weak interaction between Au and PDMS, 2 µm of parylene C(treatment with promotion solution before depositing) was deposited on the reverse side of the PDMS film with a fingerprint structure as an interlayer for bonding, and then 100 nm of Au was sputtered on top of the sensor, the pattern of the electrodes is accomplished by photolithography (a photoresist layer (AZ 4620, AZ Electronic Materials) was spin‐coated (3000 rpm, 30 s) on the Au surface, followed by prebaking at 110 °C for 5 min. After 45 s of UV exposure using a mask aligner (URE‐2000/35AL Deep UV, IOE, CAS), the photoresist was developed into a designed pattern in AZ 400k solution for 90 s. It is then wet etched and patterned by Au etchant. Rinse with acetone to remove the remaining photoresist.) (Figure S3‐III, Supporting Information). After the above steps, the gold remained firmly fixed on the PDMS surface (Figure S11, Supporting Information). The fingerprint electrode film was then obtained after demolding (Figure S3‐IV, Supporting Information).

### Preparation of the Dielectric Layer

Single‐layer GO was purchased from Graphene China as brown sheets. The average lateral particle size was ≈0.5–5.0 µm, and the thickness was ≈0.335–1.000 nm. The GO was dried in the oven at 70 °C for 12 h to remove water absorbed by hydrophilic groups on its surface. A certain weight of GO (depending on the weight percent and total mass of the final component) was added to ≈30 g of isopropanol (IPA) and magnetically stirred for 5 h to uniformly disperse the GO powder. After stirring, the required amount of PDMS polymer (SYLGARD 184 Silicone, Dow Corning) was added, and the resulting mixture was magnetically stirred at 55 °C overnight until all of the IPA had evaporated. The PDMS agent was added to give a polymer: agent ratio of 10:1, and the resulting mixture was stirred and evacuated until the fabrication of GO/PDMS was complete. Using this method, GO/PDMS with 0, 1, 3, 5, 10, and 15 wt.% of GO was fabricated (Note S3 and Figure S12, Supporting Information). The dielectric constant of the GO/PDMS hybrid material increases as the proportion of GO increases (Figure S12, Supporting Information). The microstructure of the WFES sensor's dielectric layer comprises an array of arc‐top cylindrical structures with an adjacent spacing of 200 µm. The arc‐top cylindrical structure is composed of cylinders (diameter = 100 µm, and height = 50 µm) and hemispheres (diameter = 100 µm). For the first step of dielectric layer fabrication, an arc‐top cylindrical structure mold was made using a 3D light‐curing printer, and a layer of parylene C with a thickness of 1 µm was plated onto the mold to help demolding (Figure S3‐V, Supporting Information). The PDMS was then poured on the surface of the mold (Figure S3‐VI, Supporting Information), and after curing, the arc‐top cylindrical structure on the mold was replicated, forming holes that were of the same size as the arc‐top cylindrical (Figure S3‐VII, Supporting Information). The GO/PDMS composite material with 10 wt.% of GO was spin‐coated (2,500 rpm) onto the surface of the PDMS mold (Figure S3‐VIII, Supporting Information), which was then baked in an oven at 70 °C for 12 h. In addition, a layer of parylene C with a thickness of 500 nm was deposited onto the arc‐top cylindrical structure surface (Figure S3‐IX, Supporting Information), and then the P‐GO/PDMS arc‐top cylindrical structure dielectric layer was obtained. Scanning electron microscope images and Energy‐dispersive X‐ray spectroscopies of GO/PDMS and P‐GO/PDMS dielectric layer see Figure S1 (Supporting Information).

### Preparation of the WFES Sensor

The WFES sensor consists of fingerprint electrodes, the dielectric layer, and parylene C electrodes. The parylene C electrode was made by sputtering a 100‐nm layer of Au onto a 10‐µm layer of parylene C film and then patterned by photolithography. The two electrodes and the dielectric layer were bonded together using a 5‐µm‐thick optical adhesive (acrylate adhesive) adhesive to complete the production of the WFES sensor. The total thickness of this sensor is ≈300 µm (Figure S3‐X, Supporting Information). Capacitance versus frequency curves were measured (Figure S13, Supporting Information).

### Preparation of the Graphene/polydimethylsiloxane (GO/PDMS) Hybrid Material for Dielectric Properties Test

The GO/PDMS composite material with different ratios of GO was poured into the mold and baked in an oven at 70 °C for 12 h. The solid cylindrical samples with a diameter of 1 cm and a thickness of ≈500 µm were obtained. Parylene C or parylene N of different thicknesses (0.1, 0.5, and 1 µm) were then plated on one side of the prepared 10% GO/PDMS solid samples.

### Characterization and Data Acquisition

In this work, a linear drive system powered was built by stepper motors in the x‐direction and z‐direction to apply pressure and control the relative motion between the sensor and the sample. A tensile tester (ESM 303, Mark‐10) provided mechanical pressure and pressure data acquisition. An LCR meter (HIOKI IM3570, Japan) (parallel‐capacitance mode at a sampling frequency of 10 kHz and 1 V) served as a capacitance data acquisition tool to measure the sensor's sensitivity and dielectric spectroscopy. The capacitance acquisition board (PCAP04‐EVA‐KIT, ScioSense) was used to perform sensor repeatability tests and collect high‐frequency vibrational signals generated by a cell phone and a toothbrush. The data of the surface texture recognition test was also collected by the capacitance acquisition board. All capacitance data presented in this study were obtained directly from the LCR meter or capacitance acquisition board without postprocessing. The 3D morphology of samples and sensor structures was characterized by an optical surface profilometer (Veeco/Wyko NT9300). Characterization of 2D vibrational signals of fabric samples by phase grating interferometer profilometer (Taylor Hobson, Form Talysurf Series 2). The equipment used to take SEM images and to measure the composition of the GO/PDMS and P‐GO/PDMS dielectric layers is the JSM‐5600 Scanning Electron Microscope (JEOL Japan). The GO/PDMS samples were tested in a NovoControl Concept 80 for dielectric spectroscopy at room temperature. The data plots were processed by OriginPro 2021.

## Conflict of Interest

The authors declare no conflict of interest.

## Author Contributions

S.H. and W.J.L. performed conceptualization. S.H., Q.H., C.W., M.W., C.M., K.Y., V.A.L.R., W.D., and J.W. performed methodology. S.H. and Q.H. performed an investigation. S.H., H.Z., and Q.C. performed visualization. W.J.L. performed funding acquisition. W.J.L. performed project administration. X.Y. and W.J.L. performed supervision. S.H. wrote – original draft. W.J.L., X.Y., V.A.L.R., W.D., and J.W. wrote – reviewed and edited.

## Supporting information

Supporting Information

Supplemental Movie 1

Supplemental Movie 2

## Data Availability

The data that support the findings of this study are available from the corresponding author upon reasonable request.
