# Supplementary material for: Biometric‐Tuned E‐Skin Sensor with Real Fingerprints Provides Insights on Tactile Perception: Rosa Parks Had Better Surface Vibrational Sensation than Richard Nixon
Source: Adv Sci (Weinh). 2024 Jul 10;11(34):2400234. doi: 10.1002/advs.202400234 (PMC11425864; doi:10.1002/advs.202400234)
Supplement: Supplementary file 1 — Supporting Information [file ADVS-11-2400234-s001.docx]

Supporting Information

**Biometric-Tuned E-Skin Sensor with Real Fingerprints Provides Insights on Tactile Perception: Rosa Parks Had Better Surface Vibrational Sensation than Richard Nixon**

Senlin Hou,^1, #^ Qingyun Huang,^2,3, #^ Hongyu Zhang,^1^ Qingjiu Chen,^1^ Cong Wu,^4^ Mengge Wu,^5^ Chen Meng,^1^ Kuanming Yao,^5^ Xinge Yu,^5^* Vellaisamy A. L. Roy,^6^ Walid Daoud,^1^ Jianping Wang,^7^ and Wen Jung Li,^1^*

^1^ Dept. of Mechanical Engineering, City University of Hong Kong, Kowloon, Hong Kong.

^2^ Dept. of Industrial Engineering and Management, School of Mechanical Engineering, Shanghai Jiao Tong University, Shanghai, China.

^3^ State Key Laboratory of Mechanical Systems and Vibration, Shanghai Jiao Tong University, Shanghai, China.

^4^ Hong Kong Centre for Cerebro-cardiovascular Health Engineering, Hong Kong Science Park, New Territories, Hong Kong.

^5^ Dept. of Biomedical Engineering, City University of Hong Kong, Kowloon, Hong Kong.

^6^ School of Science and Technology, Hong Kong Metropolitan University, Ho Man Tin, Hong Kong.

^7^ Dept. of Computer Science, City University of Hong Kong, Kowloon, Hong Kong.

^#^: Equal contribution authors

*: Co-contact authors

Supporting information includes:

**Supplementary Note S1:** The experimental setup for the capacitive response of the WFES sensor for rapid compression and release.

**Supplementary Note S2:** Influence of the scanning direction on the amplitude of the vibrational signal.

**Supplementary Note S3:** Selection of dielectric materials.

**Supplementary Note S4:** The sensing mechanism of the WFES sensors.

**Supplementary Note S5:** Theoretical analysis of the effects of different interridge distances on the vibrational signal.

**Supplementary Note S6:** Experimental setup for testing the pressure of leaf spring.

**Figure S1.** Scanning electron microscope (SEM) images and Energy-dispersive X-ray (EDX) spectroscopies of GO/PDMS and P-GO/PDMS dielectric layer.

**Figure S2.** SEM image (cross-sectional view) of a WFES sensor.

**Figure S3.** WFES sensor fabrication process.

**Figure S4.** Capacitance responses from a WFES sensor subjected to compressing and release at fast speeds, insets show the close-up views at the beginning and end of the test.

**Figure S5.** Experimental setup for surface texture scanning test.

**Figure S6.** Si microstrip with different interridge distances.

**Figure S7.** WFES sensor outer structure.

**Figure S8.** 3D printed microstrip with different interridge distances.

**Figure S9.** Surface profile measurement results of 7 fabrics.

**Figure S10.** Effect of scanning angle to the direction of motion on the vibrational signal.

**Figure S11.** Fingerprint film electrode patterned using photolithography.

**Figure S12.** Dielectric spectroscopy and optical images of GO/PDMS composite materials.

**Figure S13.** Capacitance versus frequency curves of WFES sensor.

**Figure S14.** The two-dimensional surface profile measurement results of sensor structures with different ridge depths and widths were obtained by the PGI profilometer.

**Figure S15.** The impact of varying ridge depths and pressure on the captured vibrational signals.

**Figure S16.** The impact of varying ridge widths on the captured vibrational signals.

**Figure S17.** Effect of sensor surface structure and pressure on contact area during scanning.

**Figure S18.** Scanning electron microscope (SEM) images of the structures of the P-GO/PDMS dielectric layer and fingerprint.

**Figure S19.** The temperature response test of the WFES sensor.

**Figure S20.** Experimental setup for testing the pressure of leaf spring.

**Table S1.** Comparison of our WFES sensor with reported sensors in terms of fabric recognition and accuracy.

**Movie S1.** Real-time tactile feedback (Manually scanning) (.MP4).

**Movie S2.** Video recording of the scanning process (.MP4).

**Supplementary Note S1:** **The experimental setup for the capacitive response of the WFES sensor for rapid compression and release.**

The WFES sensor will be affixed to the surface of the test bench with medical tape, the capacitance signal is collected using the Pcap04 capacitance acquisition board. A straight ruler is used to quickly drop one end on the sensor surface using the leverage principle, maintain it for several seconds, and then press the other end of the ruler to quickly leave the surface of the sensor. The capacitive signal response is shown in Figure S4.

**Supplementary Note S2: Influence of the scanning direction on the amplitude of the vibrational signal.**

During the test, the sample with an interridge distance of λ_s100_ was rotated by an angle θ (from 0° to 90°) to the direction of motion, and a WFES sensor with the interridge distance of λ_f450_ was scanned across the sample at the v = 1 mm/s and pressure = 2 kPa. The collected time-domain signals were filtered to remove low-frequency noise to facilitate the comparison of signal amplitudes at different scanning angles, as shown in Figure S10. From the results, the scanning direction has a great influence on the amplitude of the vibrational signal. When the scanning direction is in the range of 0-30°, the amplitude of the vibrational signal decreases with the increase of the scanning angle, and the frequency of the signal remains constant (10 Hz). However, as the scanning direction keeps increasing, when the scanning angle reaches 60°, the vibrational signal can hardly be used for surface texture recognition.

**Supplementary Note S3:** **Selection of dielectric materials.**

GO/PDMS composite materials with 0 wt%, 1 wt%, 3 wt%, 5 wt%, 10 wt%, and 15 wt% of GO were fabricated. The viscosity of the mixture decreased significantly as the weight percentage increased, the fabrication of the dielectric layer using the GO/PDMS composite was difficult, as shown in Figure S12. In this work, a 10 wt% GO/PDMS mixture was fabricated into thin films as the sensors’ dielectric layer.

**Supplementary Note S4:** **The sensing mechanism of the WFES sensors.**

The capacitance value is related to the effective dielectric constant of the dielectric layer material and the distance between electrodes.

According to the parallel plate capacitance equation (Equation. S1):

$C=\frac{\varepsilon A}{d}$ *(S1)*

Where ε is the effective dielectric constant, A is the overlap area of the plates, and d is the distance between them. And the general Lichtenecker’s mixing rule.^[1]^ (Equation. S2)

$\varepsilon={V_{0}\varepsilon}_{0}{+ V_{r}\varepsilon}_{r}$ *(S2)*

Where ε_0_ is the relative dielectric constant of air, ε_r_ is the relative dielectric constant of the dielectric material, V_0_ and V_r_ are the volume fractions (V_0_ + V_r_ = 1) of dielectric materials and air, respectively.

We used P-GO/PDMS as the sensing layer, it has a low modulus of elasticity and a high dielectric constant (ε). Additionally, the arc-top cylindrical structure in the dielectric layer provides better compression properties and higher sensitivity under lower pressures (d). When the dielectric layer is compressed by an applied force, the volume occupied by air gaps within the dielectric material will decrease as air is extruded out. Since air's dielectric constant is lower than P-GO/PDMS, the effective dielectric constant will increase.^[2-3]^ In addition, parylene C also reduces the viscoelasticity of the PDMS, allowing faster response/recovery.

**Supplementary Note S5:** **Theoretical analysis** **of the effects of different interridge distances on the vibrational signal.**

When the interridge distance of the surface roughness was significantly greater than that of the sensor’s outer structure, additional vibrations could be produced. Also, when the scanning speed and sample structure were fixed, the characteristic peaks induced by the sample structures appeared at the same frequency, but the vibration generated by the sensor structures conformed to the function ($f=\frac{v}{\lambda_{f}}$). These behaviors can be modeled as follows (Equation. S3, Equation. S4):

$f=\frac{v}{\lambda_{s}} (\lambda_{s}\leq\lambda_{f})$ *(S3)*

$f=\frac{v}{\lambda_{s}} \& \frac{v}{\lambda_{f}}(\lambda_{s}>\lambda_{f})$  *(S4)*

Where *f* is the vibration frequency, *v* is the scanning speed, and λ*_s_* is the sample’s interridge distance, λ*_f_* is the sensor structure’s interridge distance.

The characteristic peak caused by the surface roughness (λ_s900_) could be calculated: *f = v/**λ_s900_* = 15 ÷ 0.9 = 16.67 Hz.

The characteristic frequency peak of the sensor structure (λ_f220_) could be calculated: *f = v/λ_f220_* = 15 ÷ 0.22 = 68.18 Hz).

**Supplementary Note S6:** **Experimental setup for testing the pressure of leaf spring.**

Leaf spring was chosen to apply pressure because of the following considerations.

1. We want to use leaf springs to maximize the simulation of the human finger scanning process (flat contact), the leaf spring has good elasticity and can maintain a stable pressure during the sliding process (For the scanning process refer to Movie S2).

2. By choosing the material of the leaf spring and adjusting the degree of bending of the spring it is possible to control the level of pressure required.

3. In the event of a sudden change in force, the spring can be cushioned to prevent damage to the sensor.

During the test, the leaf spring was fixed to the XYZ motion stage, and a 0.2 mm thick coverslip was attached to the force gauge (Mark-10) probe to ensure stable contact between the leaf spring and the probe. The width of the leaf spring is 1 cm, which is the same as the width of the WFES sensor. The pressure exerted on the sensor surface by the leaf spring was applied by adjusting the deformation of the spring. The relationship between the deformation and pressure was pre-calibrated by the Motorized Test Stand (ESM 303) and the force gauge.


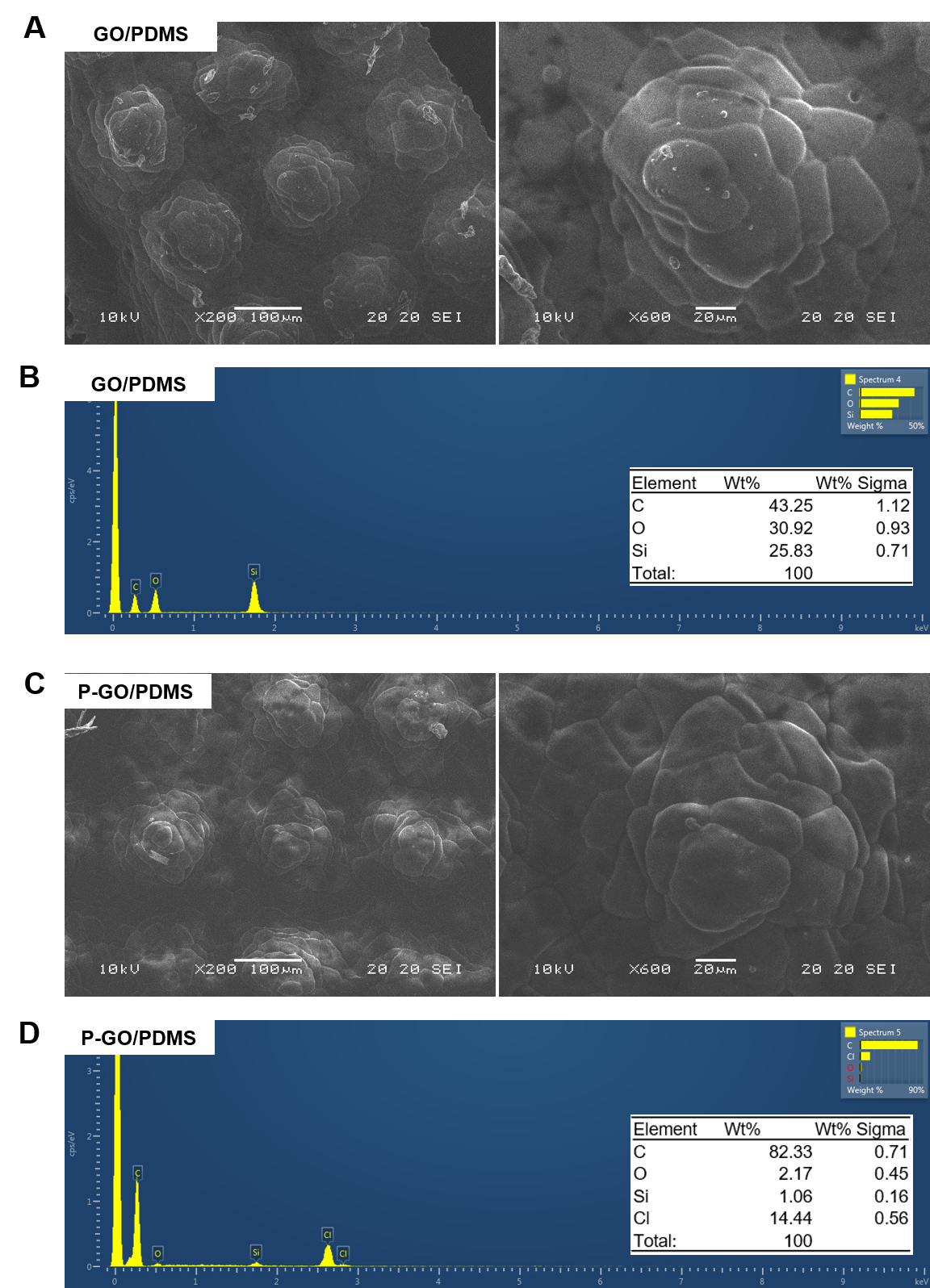


Figure S1. Scanning electron microscope (SEM) images and Energy-dispersive X-ray (EDX) spectroscopies of GO/PDMS and P-GO/PDMS dielectric layer. (A) SEM images showing the surface structure of the GO/PDMS dielectric layer. (B) EDX of GO/PDMS dielectric layer surface. (C) SEM images showing the surface structure of the P-GO/PDMS dielectric layer. (D) EDX of P-GO/PDMS dielectric layer surface.


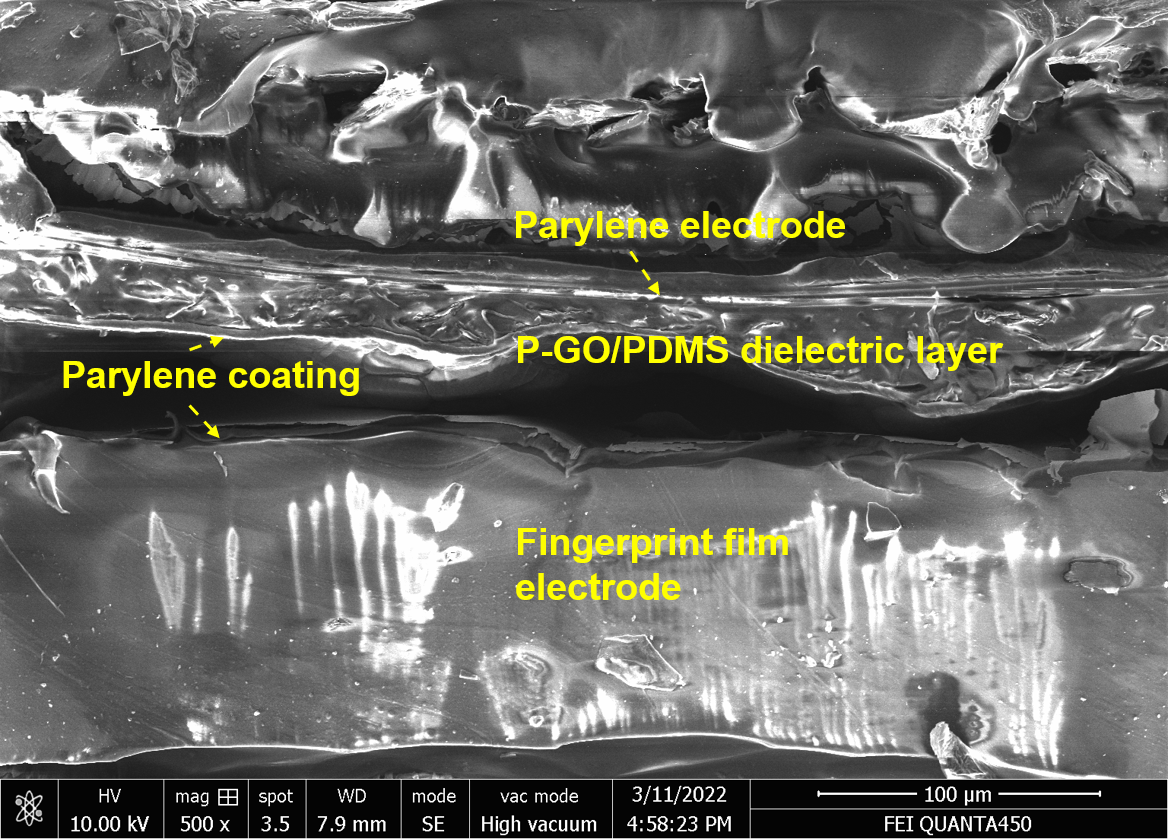


Figure S2. SEM image (cross-sectional view) of a WFES sensor.


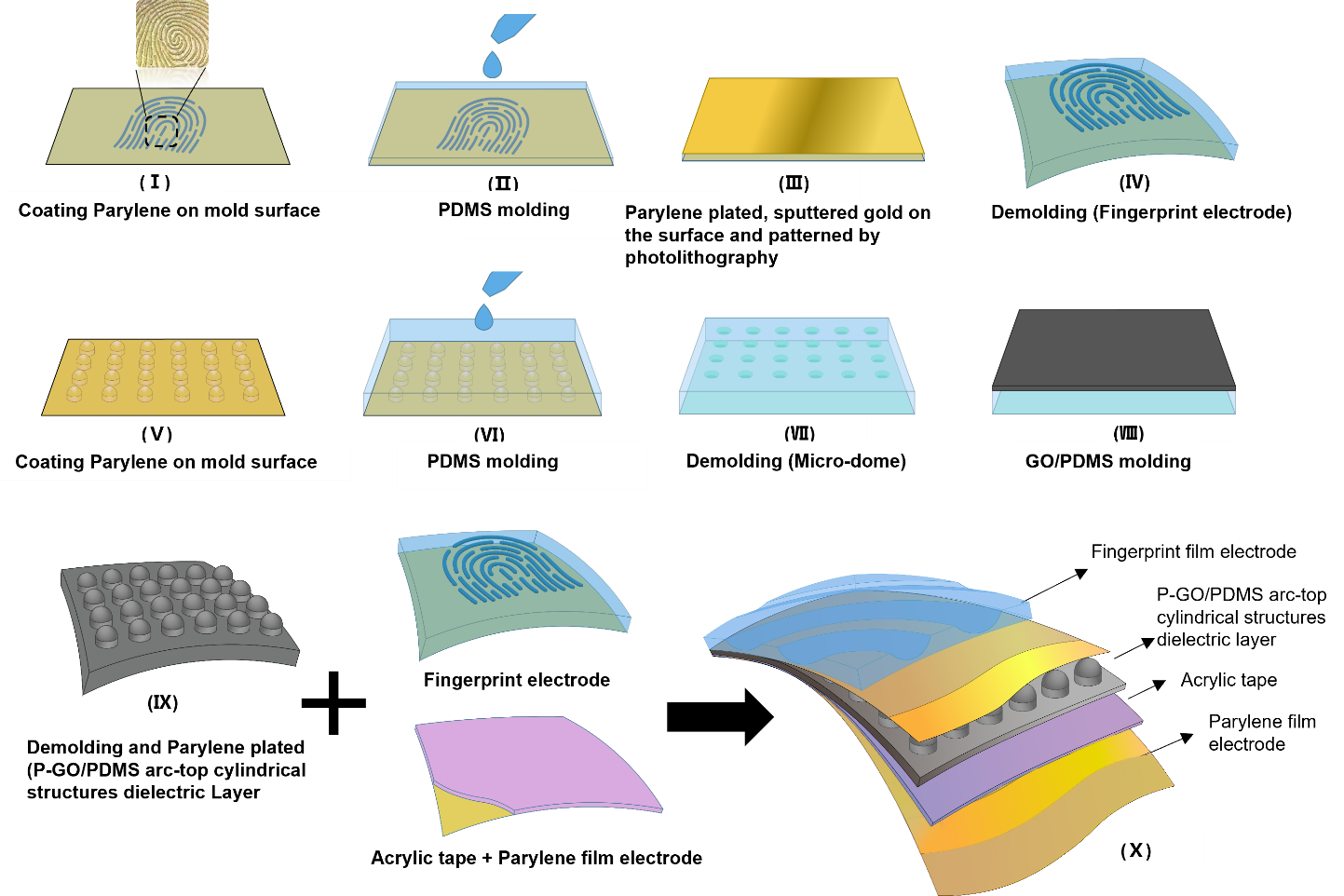


Figure S3. WFES sensor fabrication process. (Ⅰ)-(Ⅸ) Detailed processing steps for WFES sensor. (Ⅹ) Exploded view of WFES sensor.


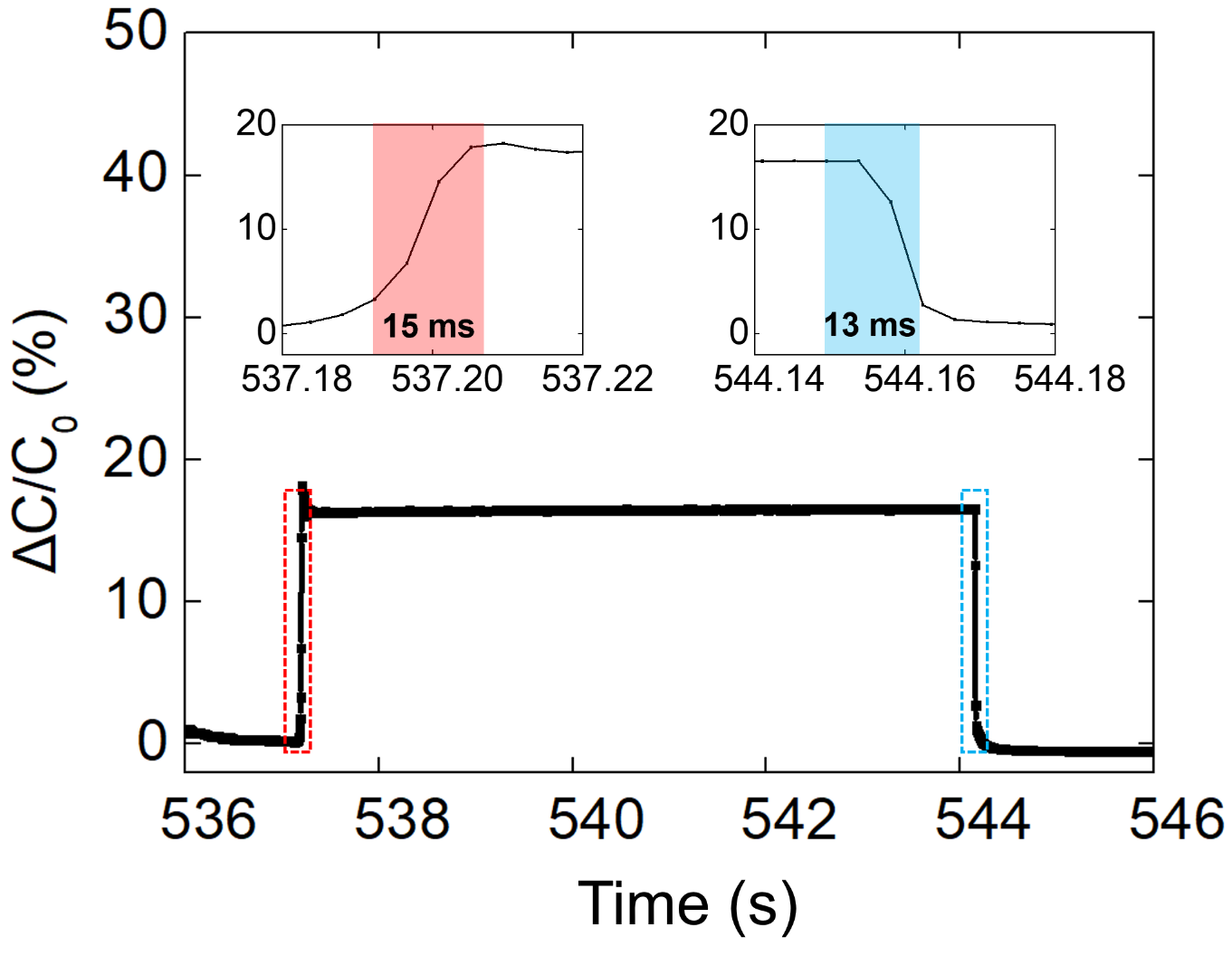


Figure S4. Capacitance responses from a WFES sensor subjected to compressing and release at fast speeds, insets show the close-up views at the beginning and end of the test.


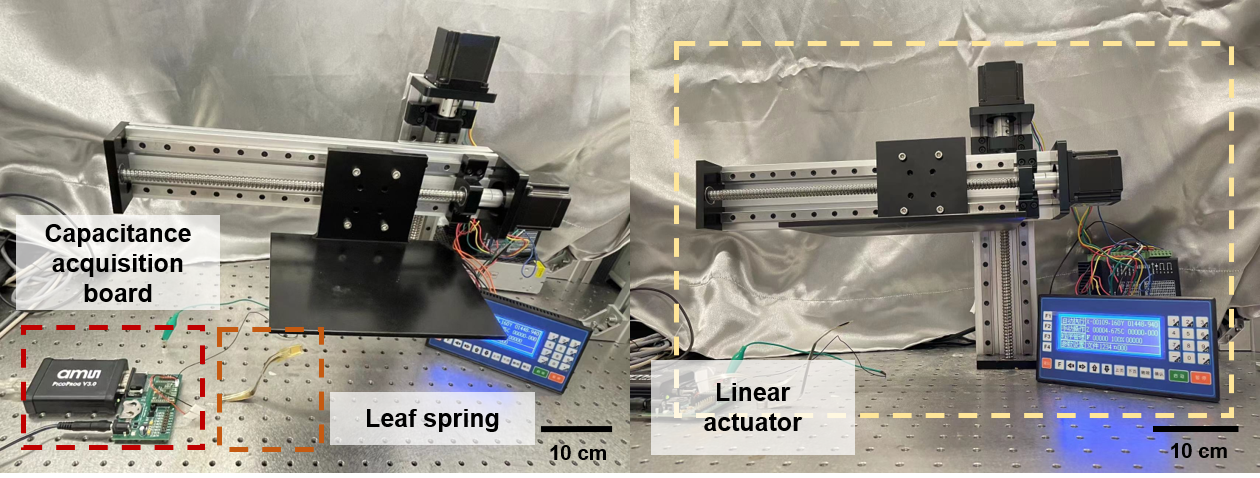


Figure S5. Experimental setup for surface texture scanning test.


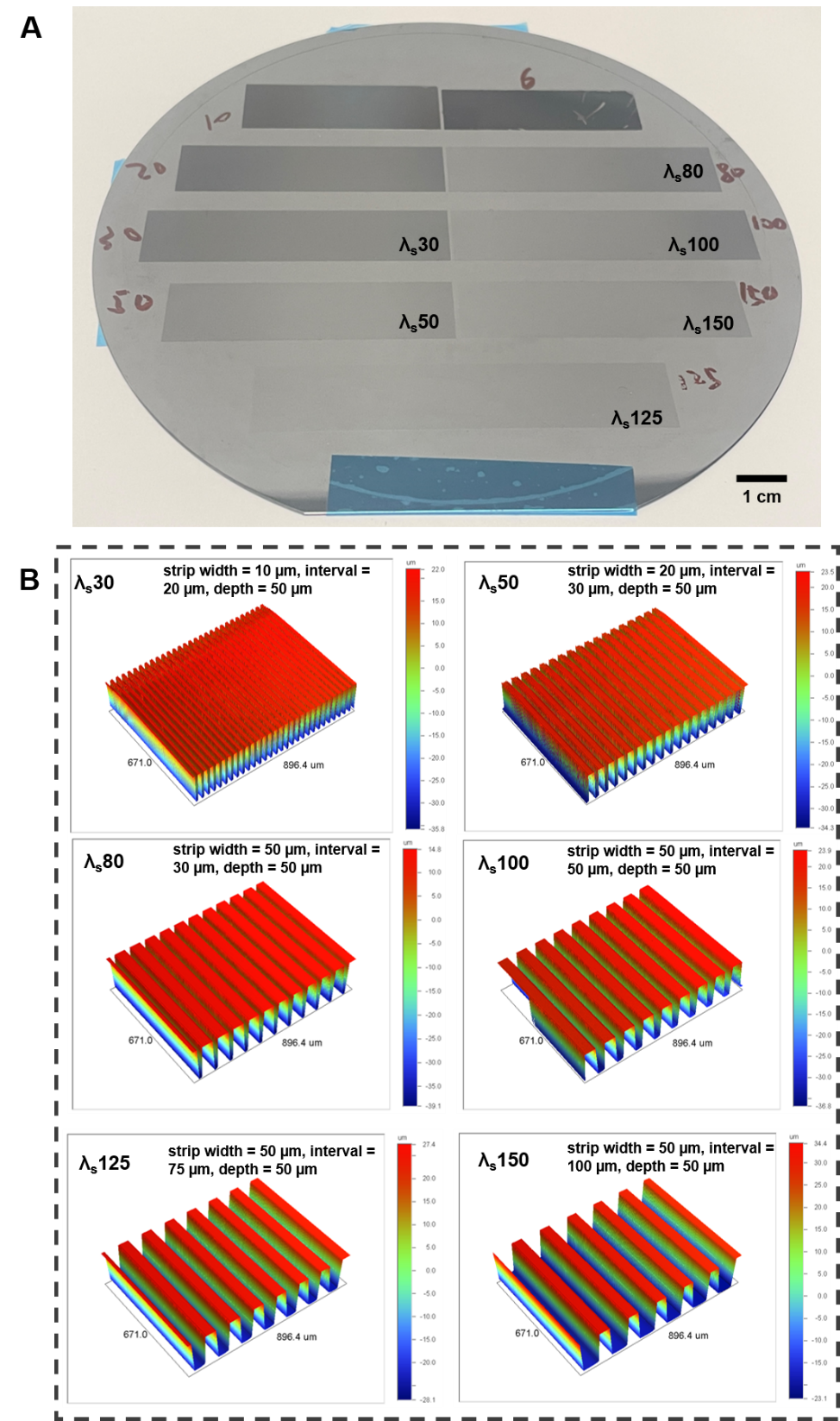


Figure S6. Si microstrip with different interridge distances. (A) Optical images of Si microstrip sample. (B) Optical 3D profile images of each Si microstrip (λ_s30,_ λ_s50,_ λ_s80,_ λ_s100,_ λ_s125,_ and λ_s150_).


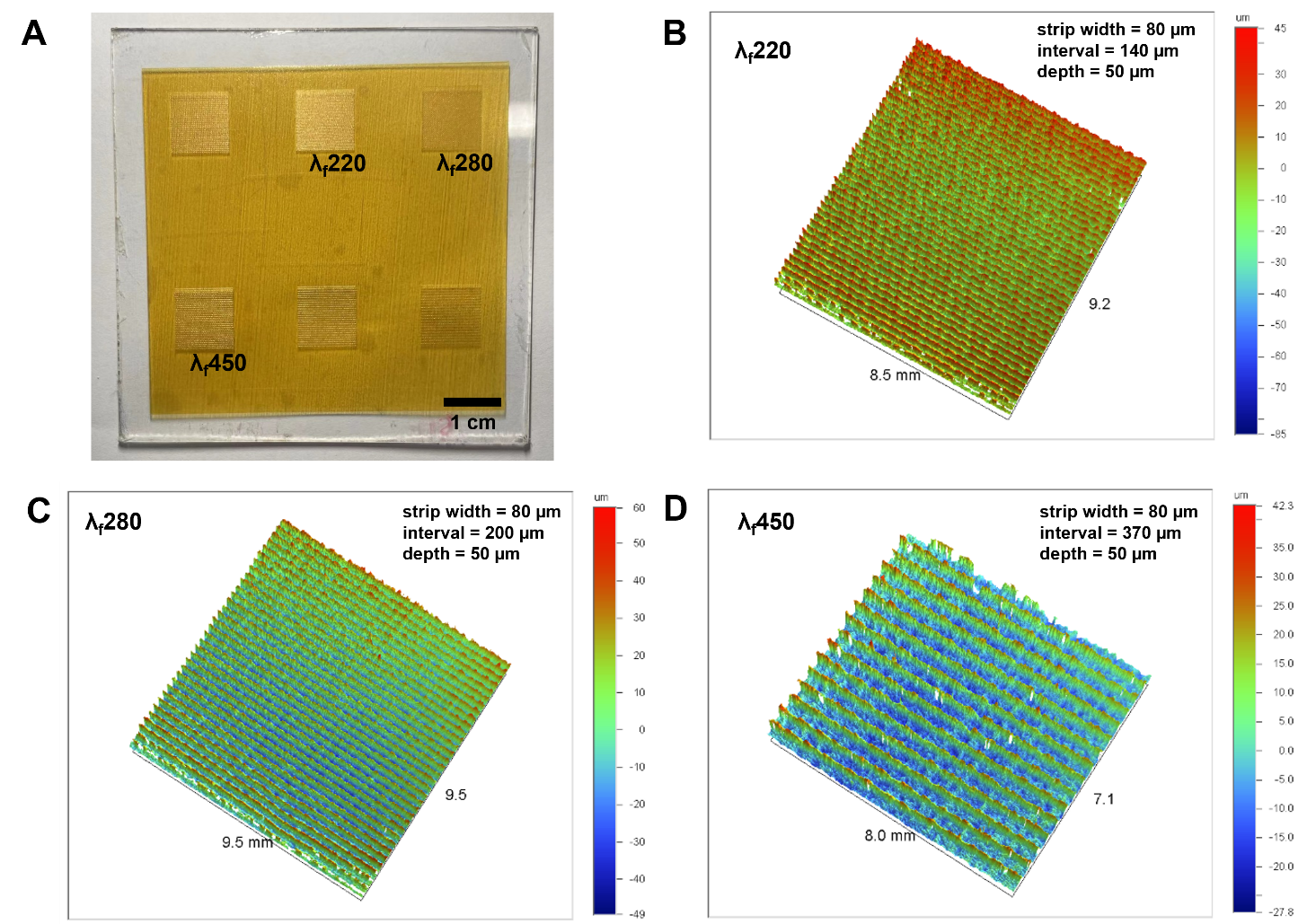


Figure S7. WFES sensor outer structure. (A) Optical images of sensor outer structure model with stripe structure. (B)-(D) Optical 3D profile images of sensor outer structure (λ_f220,_ λ_f280,_ and λ_f450_).


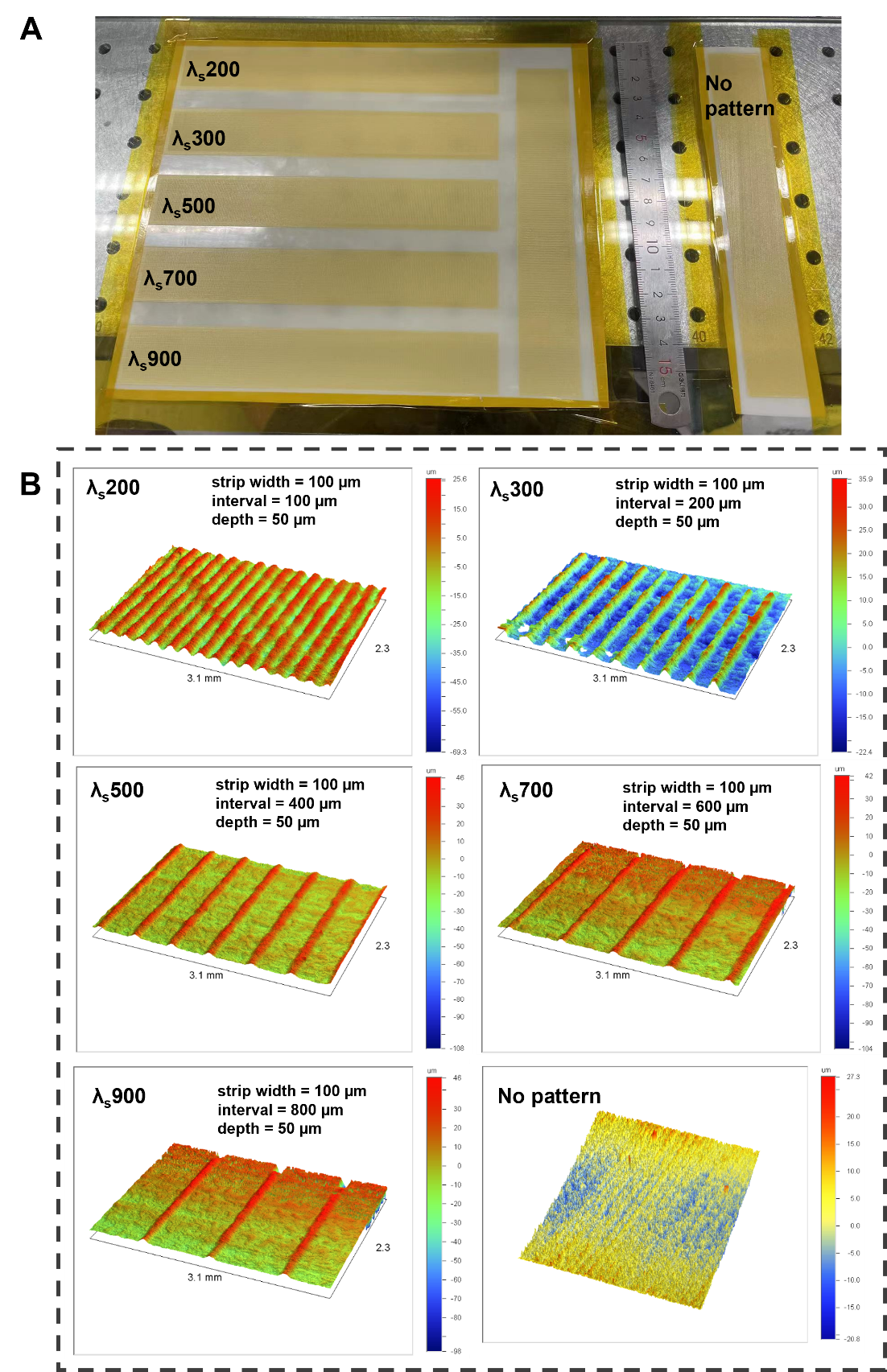


Figure S8. 3D printed microstrip with different interridge distances. (A) Optical images of 3D printed microstrip samples. (B) Optical 3D profile images of 3D printed microstrip samples (λ_s200_, λ_s300_, λ_s500_, λ_s700_, λ_s900_, and no pattern).


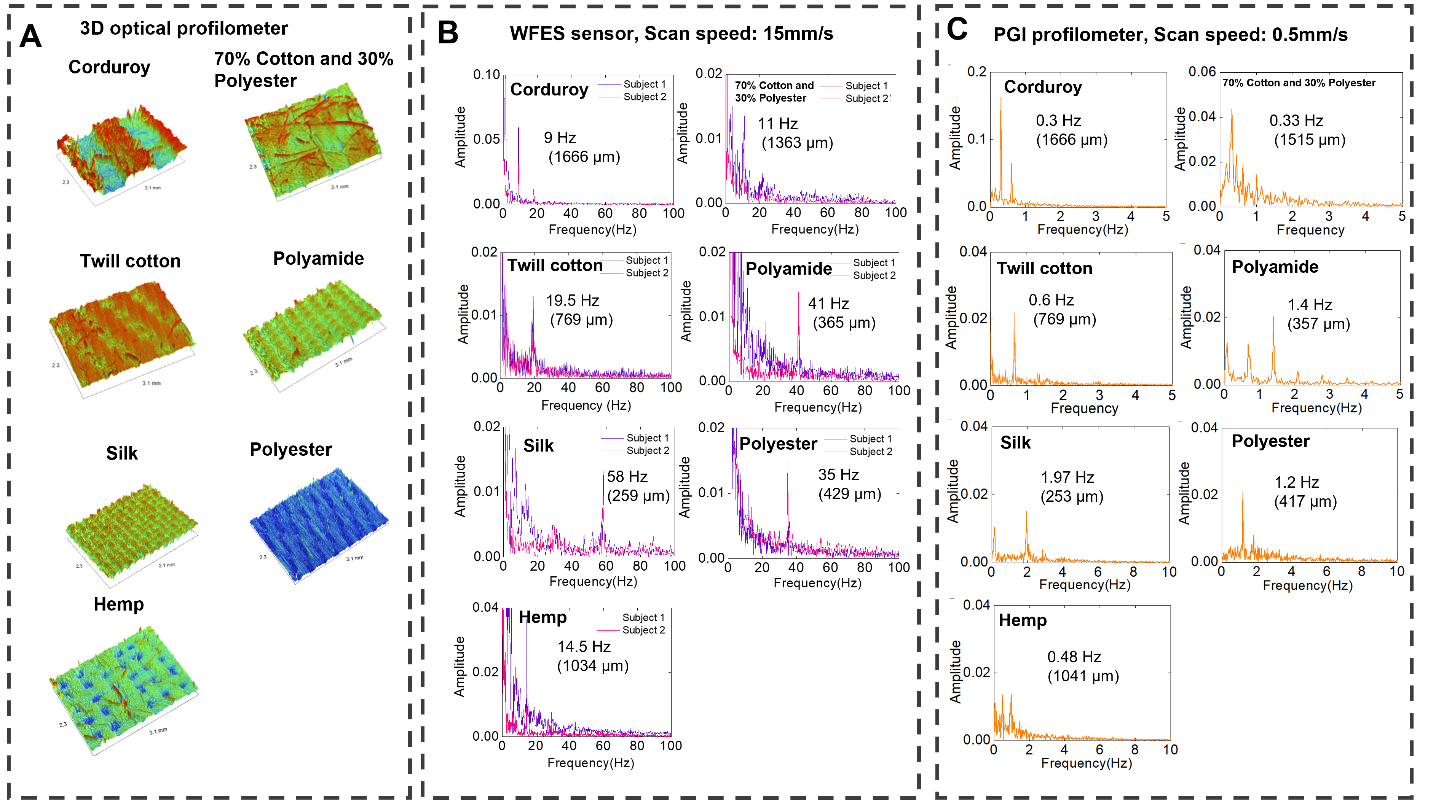


Figure S9. Surface profile measurement results of 7 fabrics. (A) The optical 3D profile images of the 7 fabrics. (B) Vibrational spectra of the 7 fabrics collected by the WFES sensor. (C) Vibrational spectra of the 7 fabrics collected by the PGI profilometer.


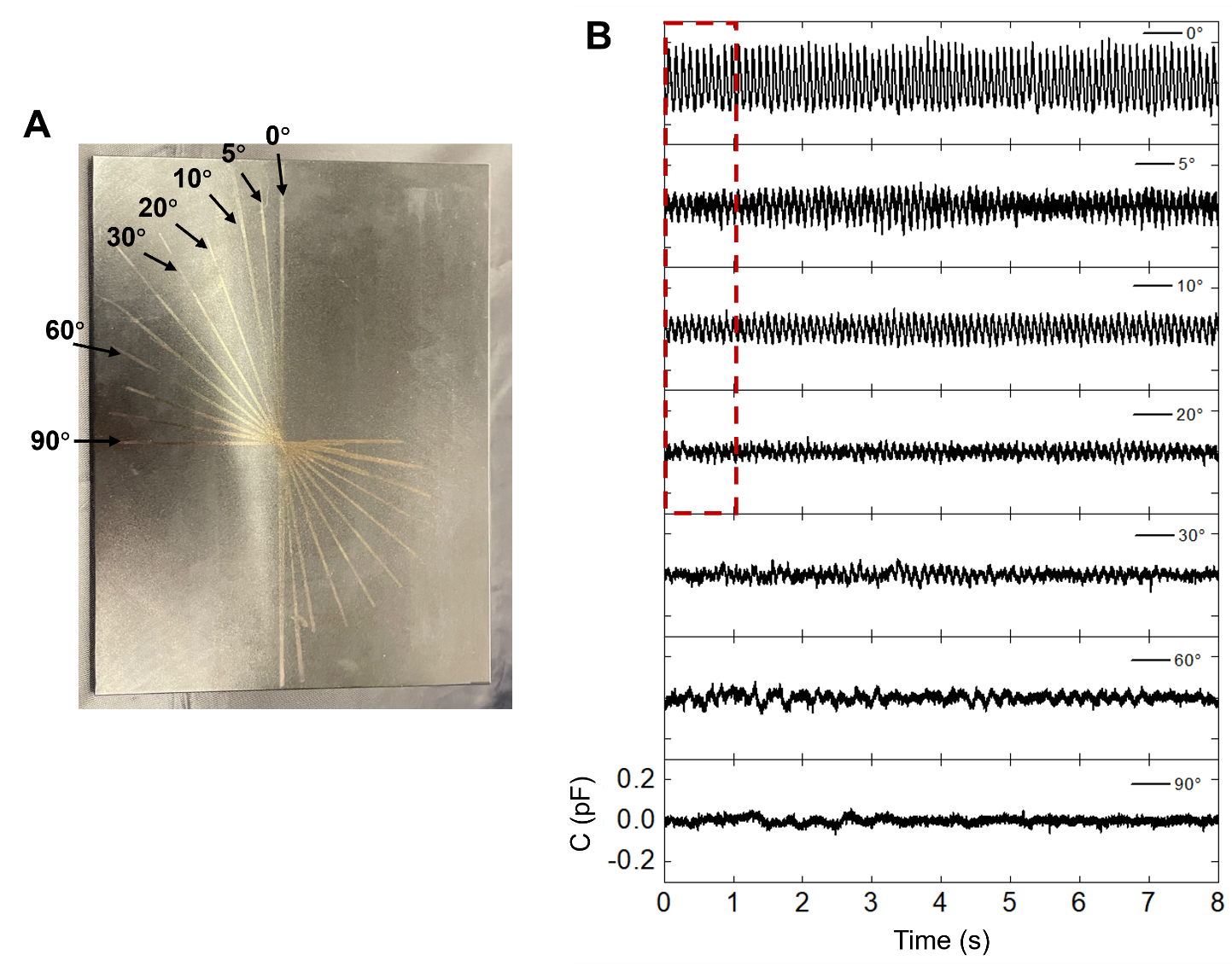


Figure S10. Effect of scanning angle to the direction of motion on the vibrational signal. (A) Linear actuator fittings marked with angular reference lines. (B) Vibrational signals were obtained at different scanning angles (angles θ from 0 to 90°, scanned sample: λ_s100_, scan speed: v = 1 mm/s, pressure = 2 kPa).


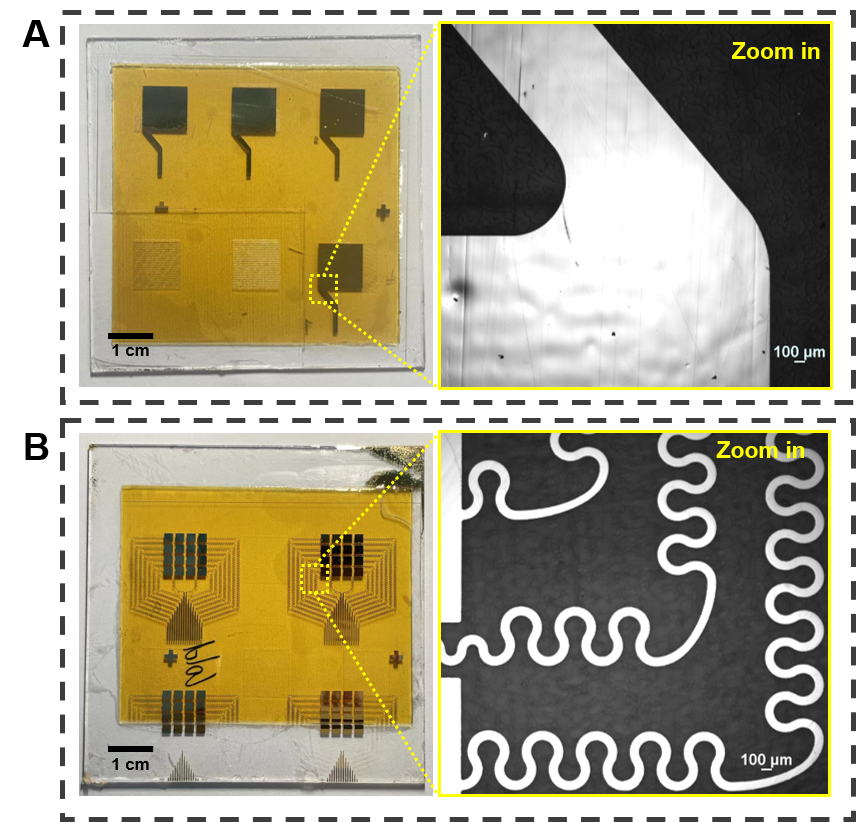


Figure S11. Fingerprint film electrode patterned using photolithography. (A) Optical images of a fingerprint electrode with a minimum line width of 1mm, the right side is a close-up view of the electrode. (B) Optical images of a fingerprint electrode with a minimum line width of 100 μm, the right side is a close-up view of the electrode.


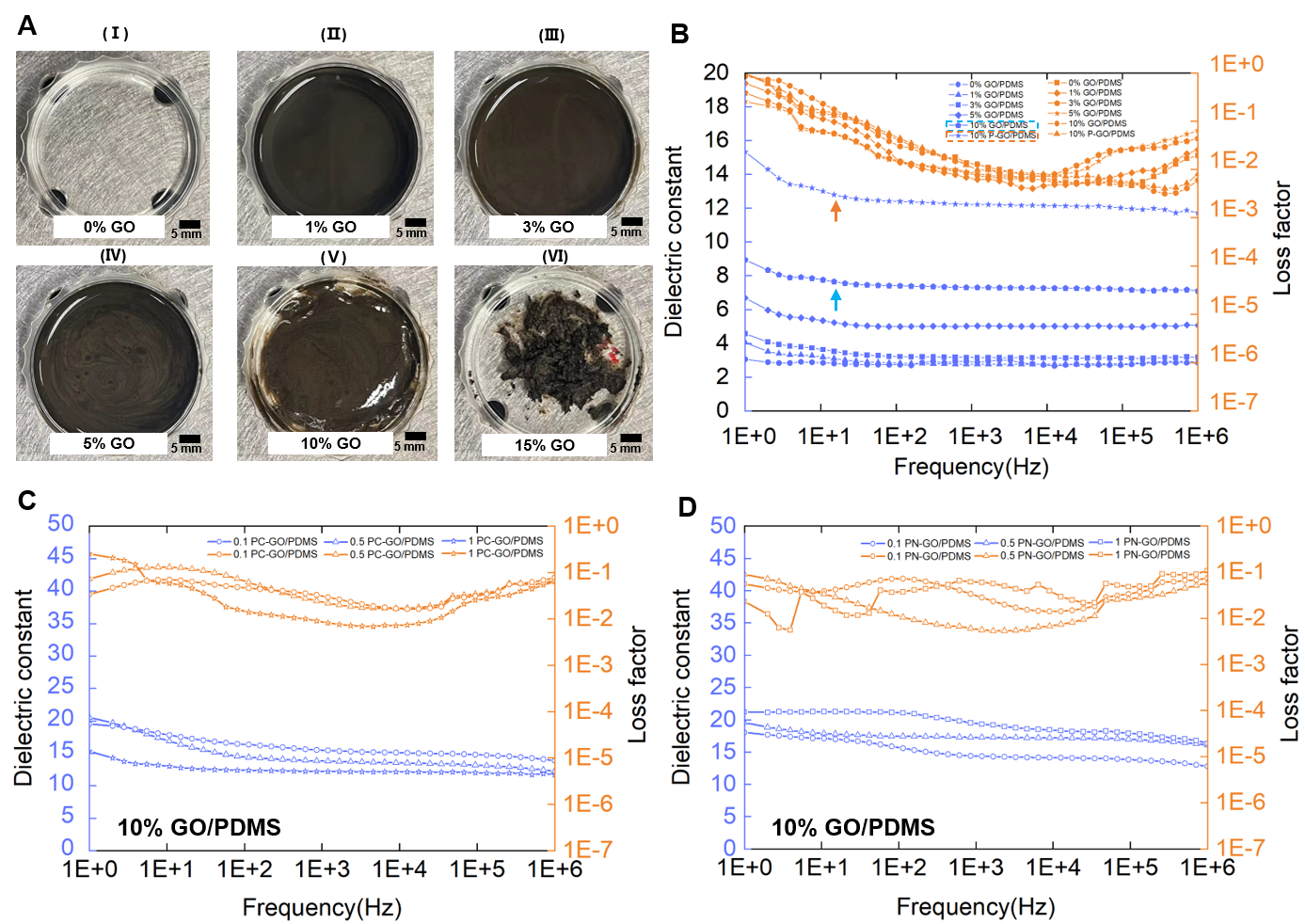


Figure S12. Dielectric spectroscopy and optical images of GO/PDMS composite materials. (A)(Ⅰ-Ⅵ) Optical images of GO/PDMS mixture with 0 wt%,1 wt%, 3 wt%, 5 wt%, 10 wt%, and 15 wt% of GO, the PDMS: PDMS agent ratio is 10:1. (B) Dielectric spectroscopy of different GO loading. (C) Dielectric spectroscopy of 10% GO/PDMS coated with parylene C with different thicknesses. (D) Dielectric spectroscopy of 10% GO/PDMS coated with parylene N with different thicknesses.


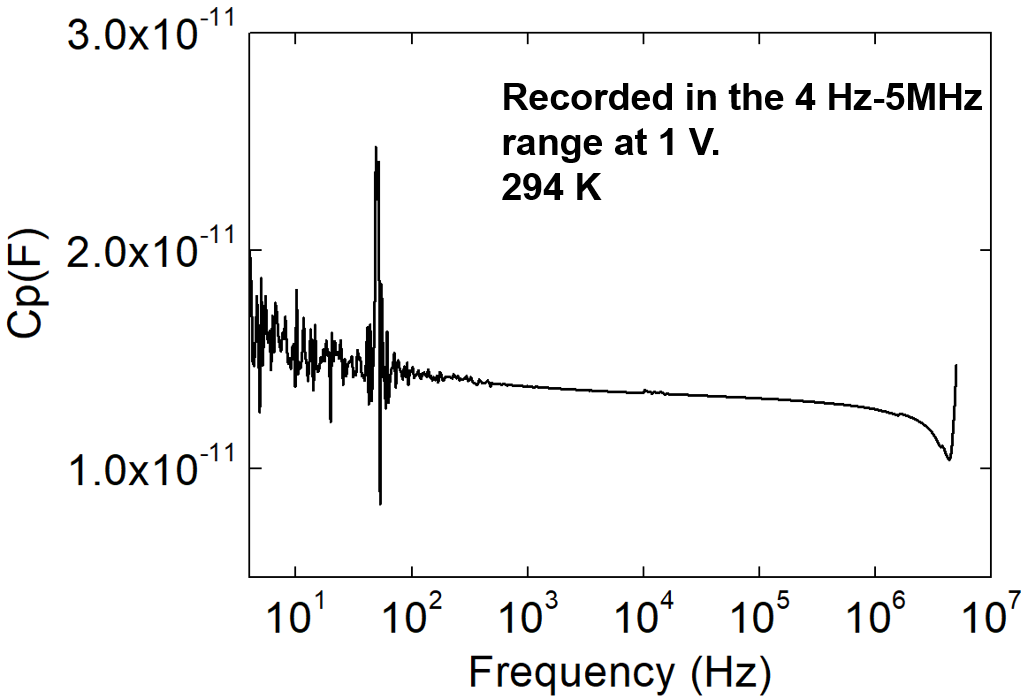


Figure S13. Capacitance versus frequency curves of WFES sensor.


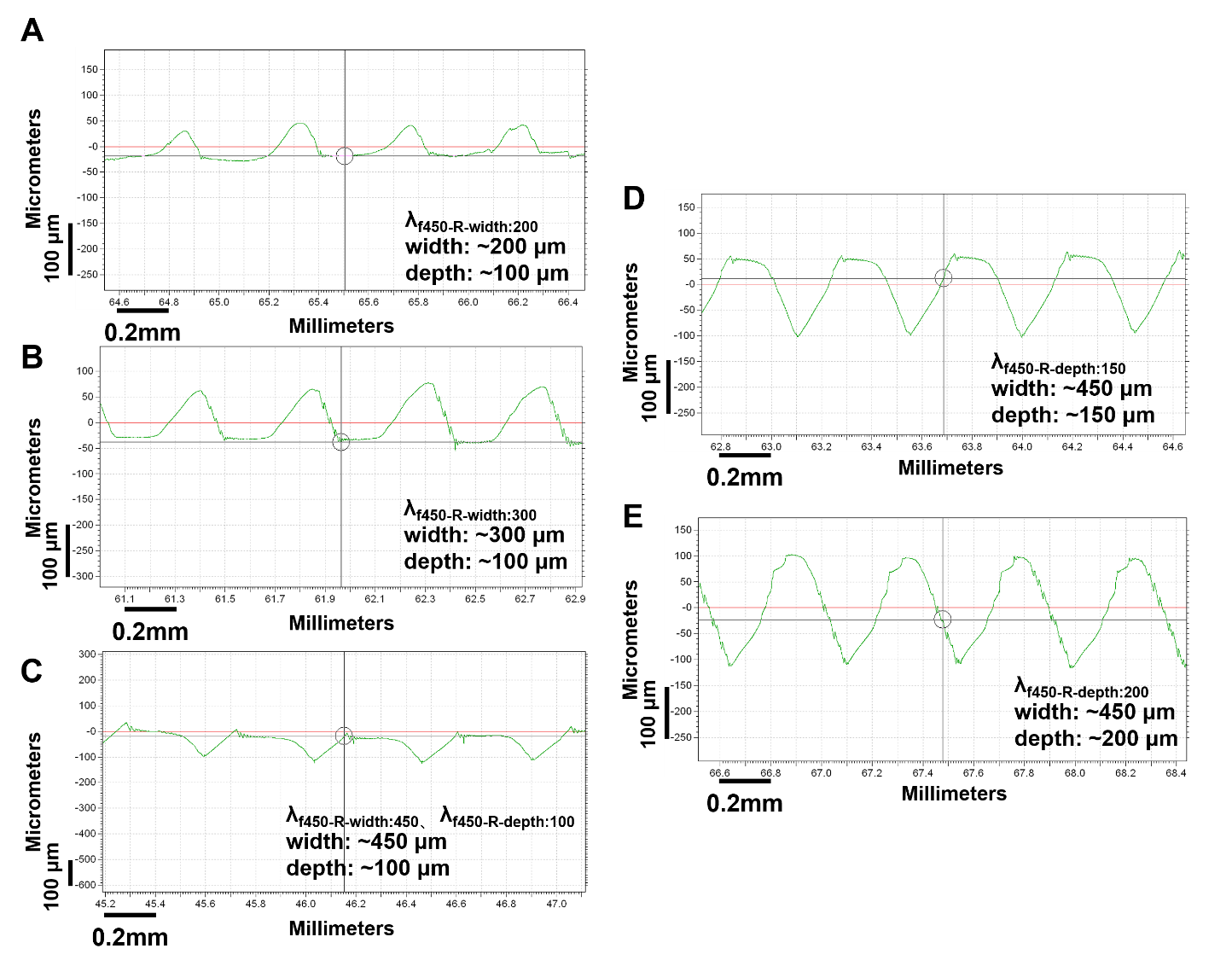


Figure S14. The two-dimensional surface profile measurement results of sensor structures with different ridge depths and widths were obtained by the PGI profilometer. (A)λ_f450-R-width:200_. (B) λ_f450-R-width:300_. (C) λ_f450-R-width:450_, λ_f450-R-depth:100_. (D) λ_f450-R-depth:150_. (E) λ_f450-R-depth:200_.


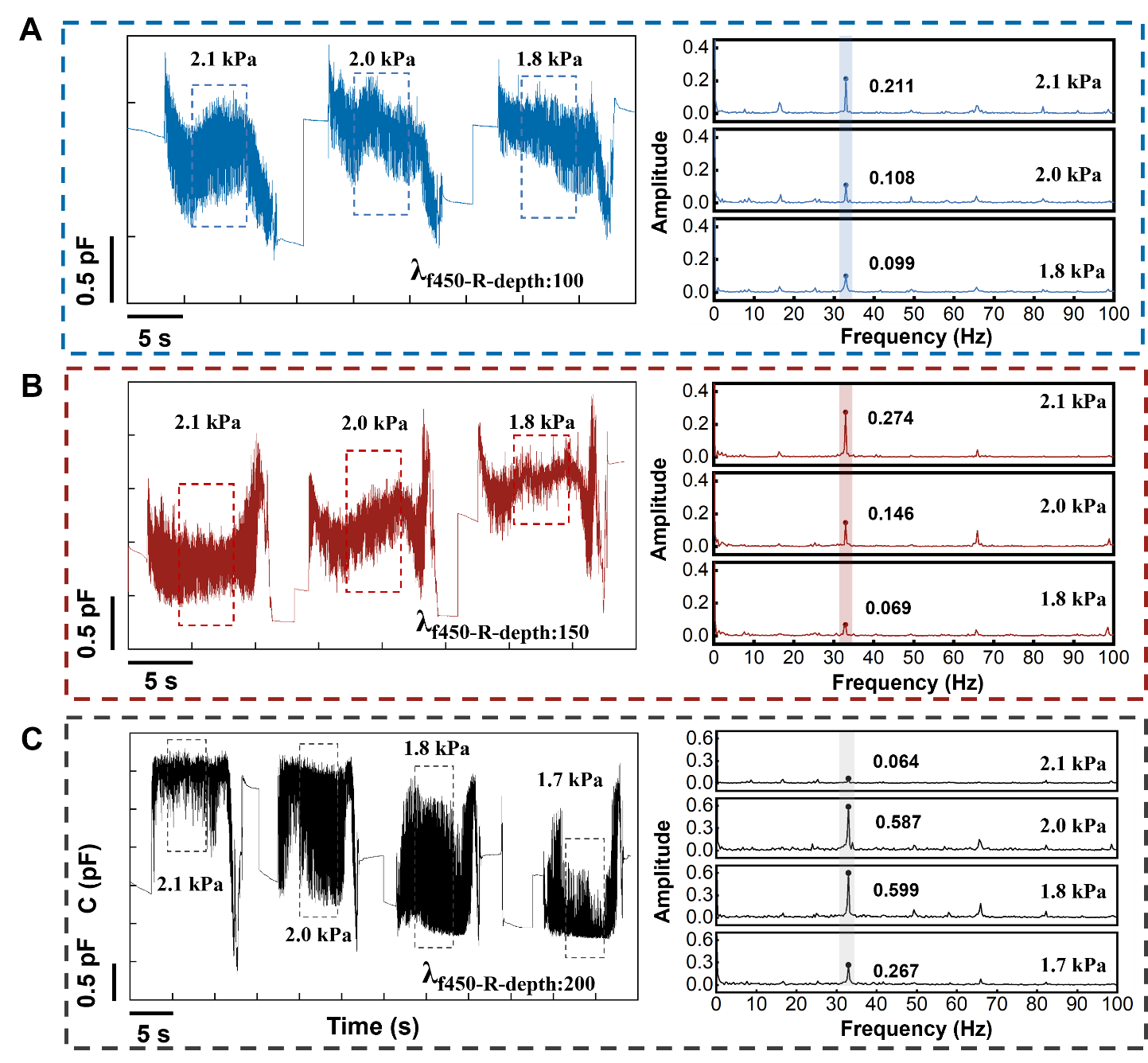


Figure S15. The impact of varying ridge depths and pressure on the captured vibrational signals. (A) Vibrational signal and spectra were acquired by the sensor with ridge depth around 100 μm (λ_f450-R-depth:100_) by scanning the microstrip at a speed of 15 mm/s. (B) Vibrational signal and spectra were acquired by the sensor with ridge depth around 150 μm (λ_f450-R-depth:150_) by scanning the microstrip at a speed of 15 mm/s. (C) Vibrational signal and spectra were acquired by the sensor with ridge depth around 200 μm (λ_f450-R-depth:200_) by scanning the microstrip at a speed of 15 mm/s.


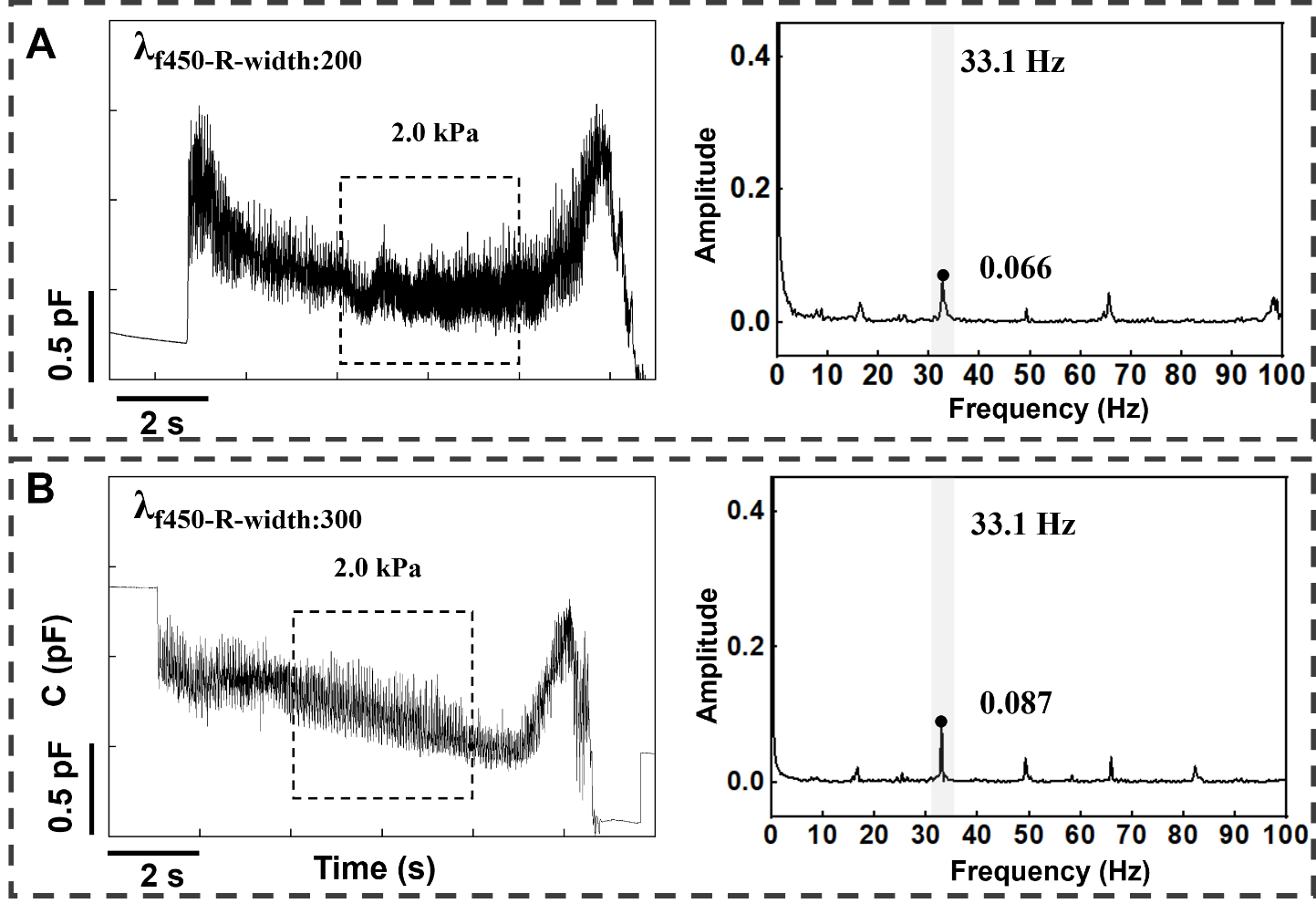


Figure S16. The impact of varying ridge widths on the captured vibrational signals. (A) Vibrational signal and spectra were acquired by the sensor with a ridge width of around 200 μm (λ_f450-R-depth:200_) by scanning the microstrip at a speed of 15 mm/s. (B) Vibrational signal and spectra were acquired by the sensor with a ridge width of around 300 μm (λ_f450-R-depth:300_) by scanning the microstrip at a speed of 15 mm/s.


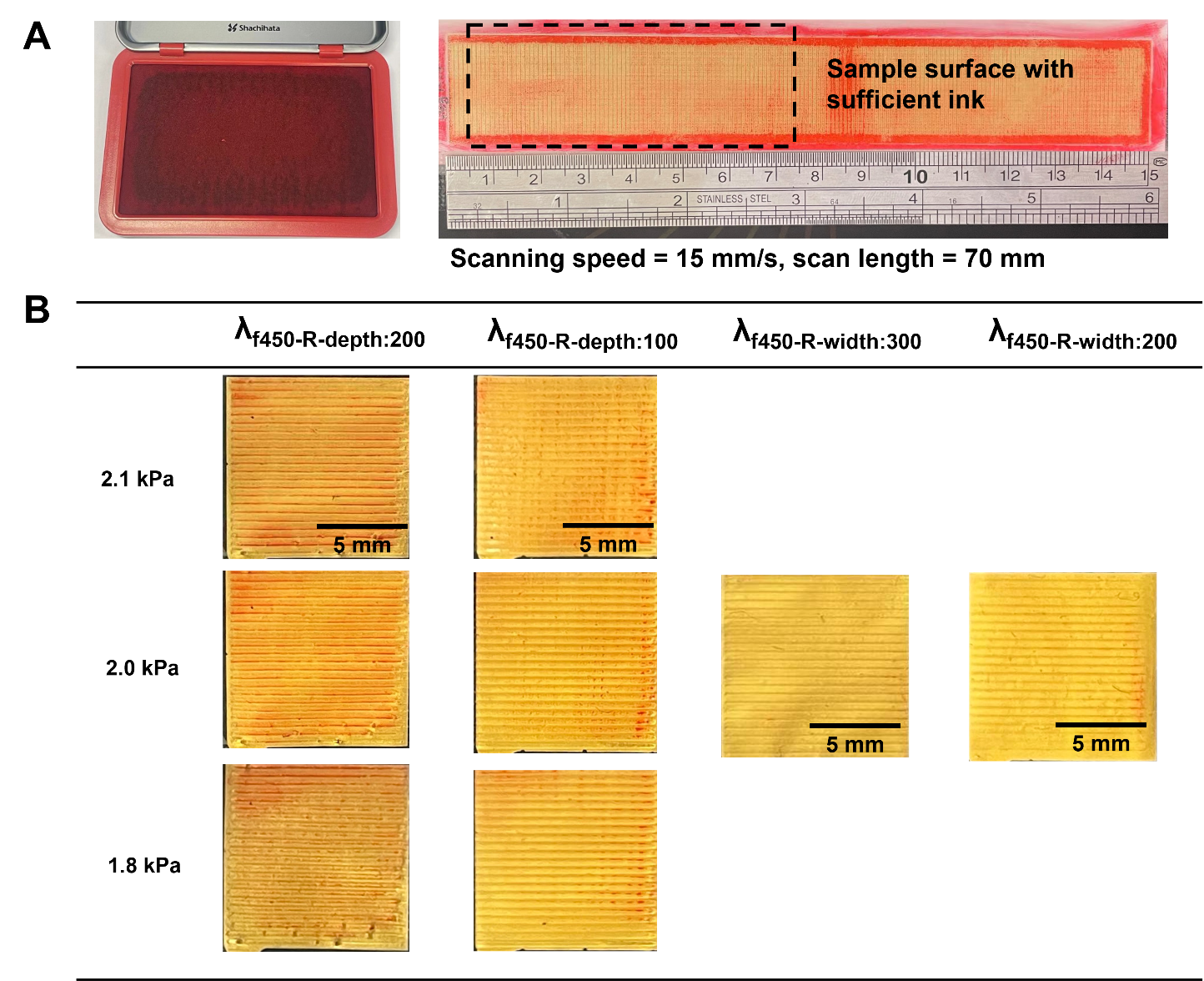


Figure S17. Effect of sensor surface structure and pressure on contact area during scanning. (A) Optical images of the ink and the sample surface after uniformly applying sufficient ink. (B) Ink staining test results of the sensor surface after scanning a 70 mm sample length at a scanning speed of 15 mm/s.


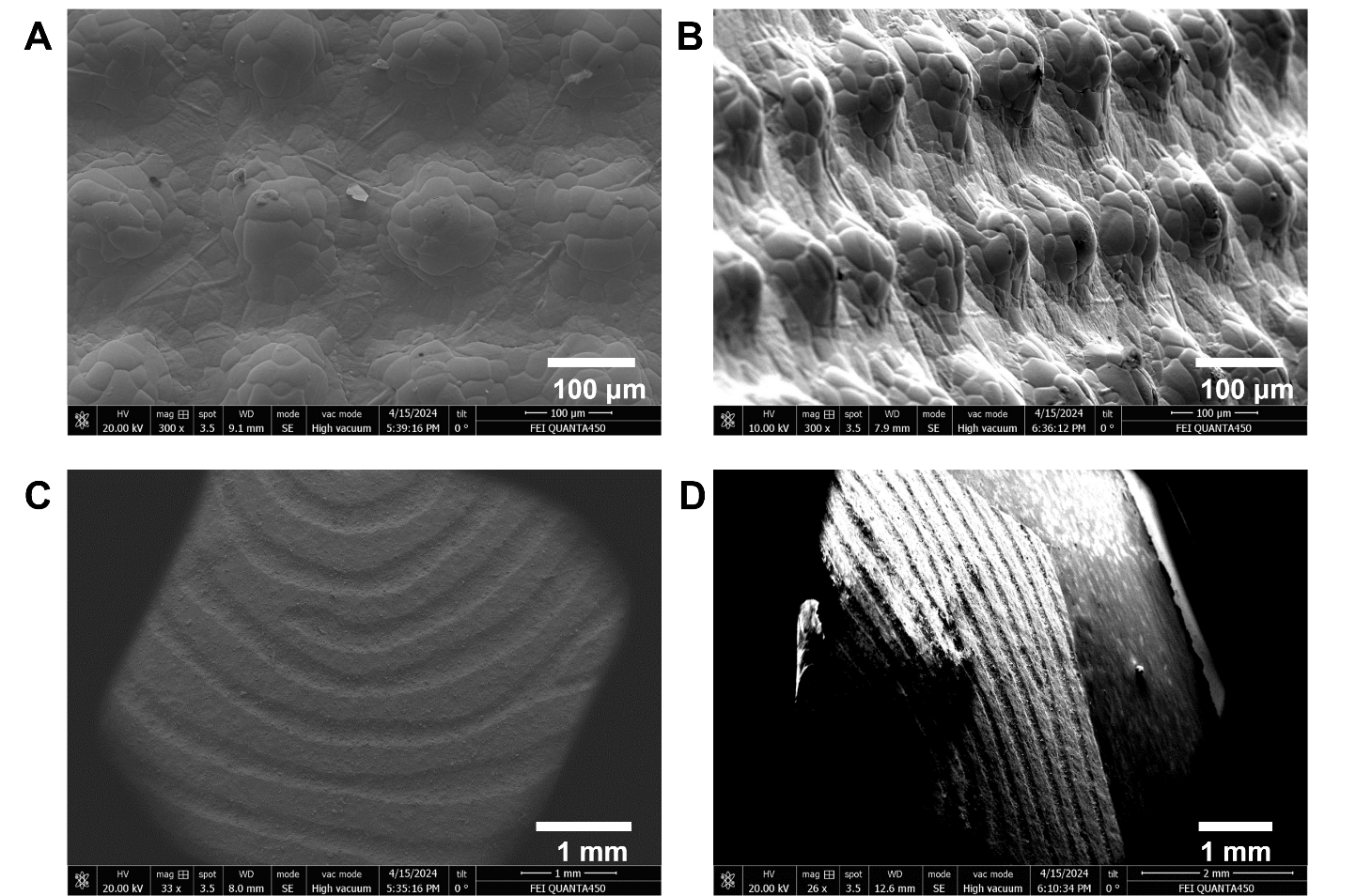


Figure S18. Scanning electron microscope (SEM) images of the structures of the P-GO/PDMS dielectric layer and fingerprint. (A) Top-view of the surface structure of the P-GO/PDMS dielectric layer. (B) Side view of the surface structure of the P-GO/PDMS dielectric layer obtained after slanting the angle of 45 degrees. (C) Top-view of the surface structure of the fingerprint structure. (D) Side view of the fingerprint structure obtained after slanting the angle of 45 degrees.


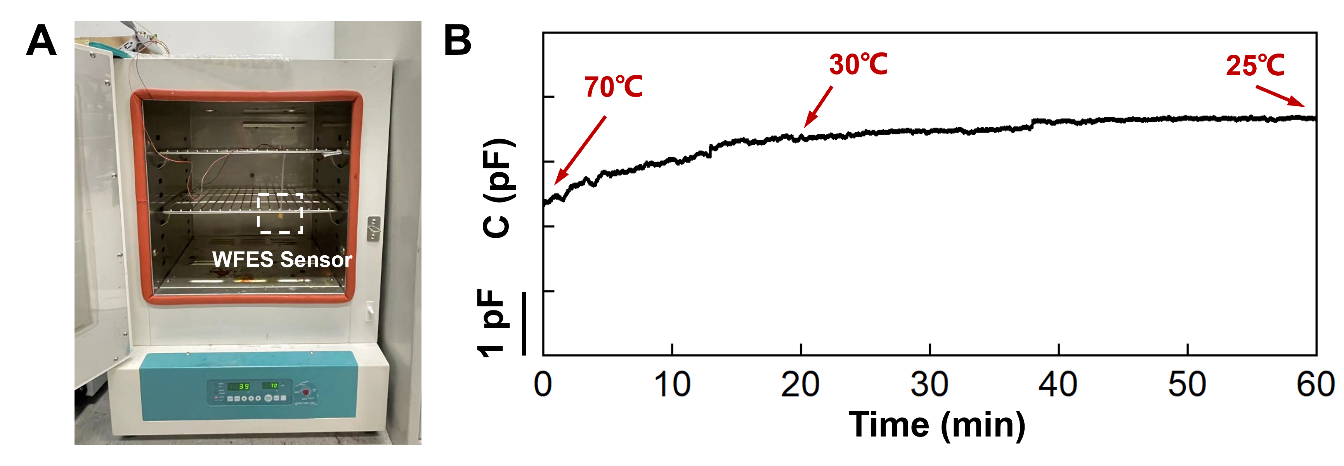


Figure S19. The temperature response test of the WFES sensor. (A) Experimental setup for testing the temperature response test. (B) Result of the temperature response test.


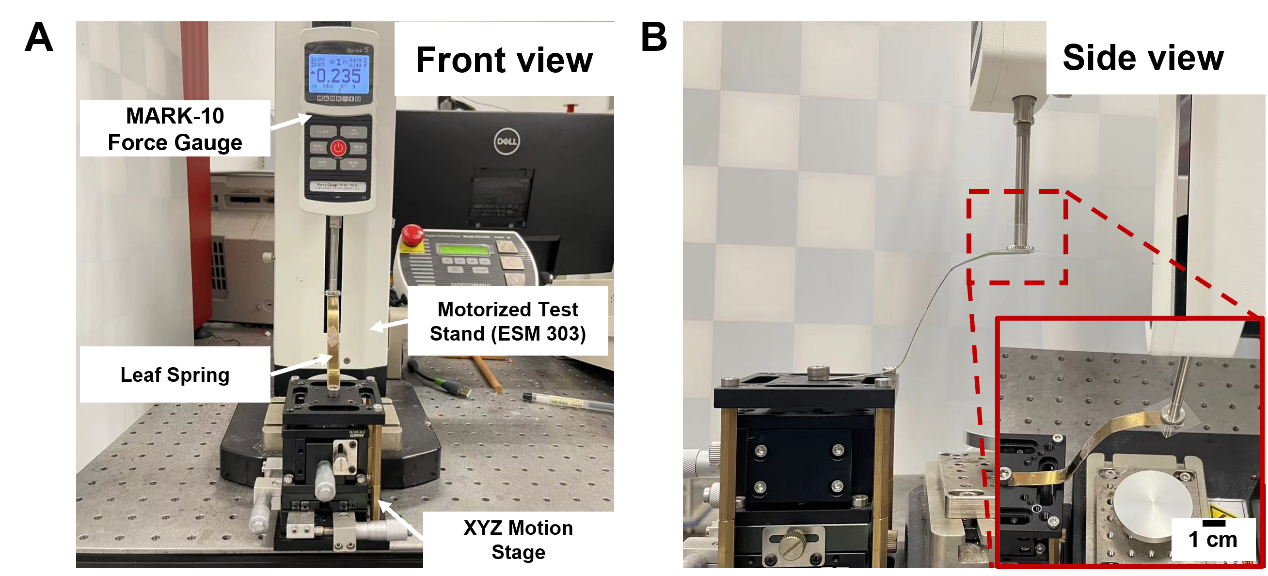


Figure S20. Experimental setup for testing the pressure of leaf spring. (A) Optical images of the front view of the experiment setup. (B) Optical images of the side view of the experiment setup, inlet show the close-up view at the contact position of leaf spring and force gauge.

Table S1. Comparison of our WFES sensor with reported sensors in terms of surface texture recognition and accuracy.^[4-11]^


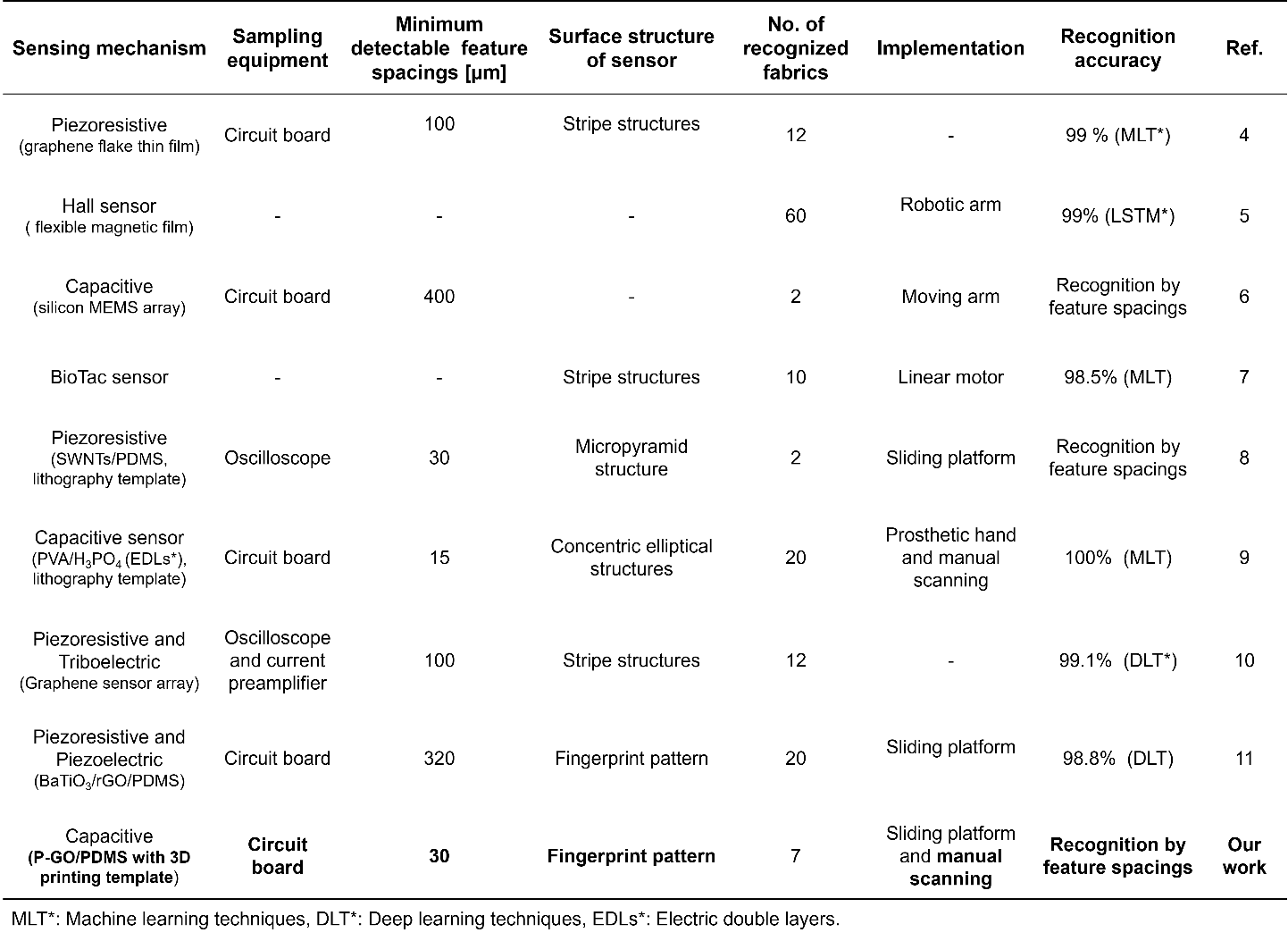


Movie S1.

Real-time tactile feedback, where the finger wearing the WFES sensor gets vibration signals of different frequencies when it scans microstrip with different interridge distances. Left top: finger scanning video recording. Left bottom: screen recording of the GUI of PCAP04-EVA-KIT. Right: close-up of real-time capacitive signal output.

Movie S2.

Video recording of the scanning process. A sensor with an interridge distance of λ_f450_ was used to scan the surface of the sample with an interridge distance of λ_s900_ at a scanning speed of 15 mm/s and a pressure of 2 kPa.

**References**

[1] Y. Wu, X. Zhao, F. Li, Z. Fan, *J. Electroceram.* **2003**, *11* (3), 227.

[2] S. C. Mannsfeld, B. C. Tee, R. M. Stoltenberg, C. V. Chen, S. Barman, B. V. Muir, A. N. Sokolov, C. Reese, Z. Bao, *Nat Mater* **2010**, *9* (10), 859.

[3] H. Niu, H. Zhang, W. Yue, S. Gao, H. Kan, C. Zhang, C. Zhang, J. Pang, Z. Lou, L. Wang, Y. Li, H. Liu, G. Shen, *Small* **2021**, *17* (41), 2100804.

[4] S. Chun, I. Hwang, W. Son, J.-H. Chang, W. Park, *Nanoscale* **2018**, *10* (22), 10545.

[5] Y. Yan, Z. Hu, Y. Shen, J. Pan, *Adv. Intell. Syst.* **2022**, *4* (1), 2100076.

[6] H. B. Muhammad, C. Recchiuto, C. M. Oddo, L. Beccai, C. J. Anthony, M. J. Adams, M. C. Carrozza, M. C. L. Ward, *Microelectron. Eng.* **2011**, *88* (8), 1811.

[7] S. Huang, H. Wu, *Sensors* **2021**, *21* (15), 5224.

[8] Y. Cao, T. Li, Y. Gu, H. Luo, S. Wang, T. Zhang, *Small* **2018**, *14* (16), 1703902.

[9] N. Bai, Y. Xue, S. Chen, L. Shi, J. Shi, Y. Zhang, X. Hou, Y. Cheng, K. Huang, W. Wang, J. Zhang, Y. Liu, C. F. Guo, *Nat. Commun.* **2023**, *14* (1).

[10] S. Chun, W. Son, H. Kim, S. K. Lim, C. Pang, C. Choi, *Nano Lett.* **2019**, *19* (5), 3305.

[11] S. Chun, J.-S. Kim, Y. Yoo, Y. Choi, S. J. Jung, D. Jang, G. Lee, K.-I. Song, K. S. Nam, I. Youn, D. Son, C. Pang, Y. Jeong, H. Jung, Y.-J. Kim, B.-D. Choi, J. Kim, S.-P. Kim, W. Park, S. Park, *Nat. Electron.* **2021**, *4* (6), 429.
